# Variation for Nitrogen Use Efficiency Traits in Wheat Under Contrasting Nitrogen Treatments in South-Eastern Europe

**DOI:** 10.3389/fpls.2021.682333

**Published:** 2021-11-18

**Authors:** Marko Ivić, Sonja Grljušić, Ivana Plavšin, Krešimir Dvojković, Ana Lovrić, Bruno Rajković, Marko Maričević, Marko Černe, Brigita Popović, Zdenko Lončarić, Alison R. Bentley, Stéphanie M. Swarbreck, Hrvoje Šarčević, Dario Novoselović

**Affiliations:** ^1^Agricultural Institute Osijek, Osijek, Croatia; ^2^Centre of Excellence for Biodiversity and Molecular Plant Breeding (CroP-BioDiv), Zagreb, Croatia; ^3^Faculty of Agriculture Zagreb, University of Zagreb, Zagreb, Croatia; ^4^BC Institute for Breeding and Production of Field Crops Zagreb, Rugvica, Croatia; ^5^Institute for Agriculture and Tourism Poreč, Poreč, Croatia; ^6^Faculty of Agrobiotechnical Sciences, University of Josip Juraj Strossmayer in Osijek, Osijek, Croatia; ^7^International Maize and Wheat Improvement Center (CIMMYT), Texcoco, Mexico; ^8^The John Bingham Lab, National Institute of Agricultural Botany (NIAB), Cambridge, United Kingdom

**Keywords:** *Triticum aestivum*, wheat breeding, nitrogen use efficiency, genetic variances and correlations, heritability, indirect selection, genetic progress

## Abstract

Wheat cultivars differ in their response to nitrogen (N) fertilizer, both in terms of its uptake and utilization. Characterizing this variation is an important step in improving the N use efficiency (NUE) of future cultivars while maximizing production (yield) potential. In this study, we compared the agronomic performance of 48 diverse wheat cultivars released between 1936 and 2016 at low and high N input levels in field conditions to assess the relationship between NUE and its components. Agronomic trait values were significantly lower in the low N treatment, and the cultivars tested showed a significant variation for all traits (apart from the N remobilization efficiency), indicating that response is genotype-dependent, although significant genotype × environment effects were also observed. Overall, we show a varietal improvement in NUE over time of 0.33 and 0.30% year^–1^ at low and high N, respectively, and propose that this is driven predominantly by varietal selection for increased yield. More complete understanding of the components of these improvements will inform future targeted breeding and selection strategies to support a reduction in fertilizer use while maintaining productivity.

## Introduction

Climate change, competition for land, limited natural resources, and the co-occurrence of abiotic and biotic stresses all threaten global wheat production. One option to address this is increasing productivity through the adoption of cultivars with improved genetic potential (Reynolds et al., 2012). Over the last 50 years, breeding and agronomic efforts have led to a reported tripling of cereal yields (Pingali, 2012). However, wheat productivity is increasing at a global rate of 1.1% per year (Dixon et al., 2009) against a predicted demand requirement of 1.7% year^–1^ until 2050 (Rosegrant and Agcaoili, 2010).

Current wheat production demands a range of agrochemical inputs, including nitrogen (N) fertilizer. Between 85 and 90 million tons of N fertilizers are added to agricultural soils worldwide each year, and current predictions based on projected food demand show that this could increase to 240 million tons by 2050 (Good et al., 2004). N fertilizer represents the single most expensive input into wheat production and accounts for more than 70% of its associated greenhouse gas emissions (Mortimer et al., 2004). Excessive usage has a range of negative impacts on the environment, and it is estimated that 50−70% of applied N is lost from the plant–soil system through a combination of leaching, surface run-off, denitrification, volatilization, and microbial consumption (Peoples et al., 1995).

Plants use N as part of many biological processes (Masclaux-Daubresse et al., 2008), and it is taken up primarily as nitrate and ammonium, with nitrate being the predominant form in most agricultural soils (Crawford and Forde, 2002). Several physiological and biochemical changes occur in plants as adaptive responses to N limitation, including an increase in N uptake by high-affinity transporters, remobilization of N from older to younger leaves and reproductive parts, retardation of growth and photosynthesis, and increased anthocyanin accumulation (Ono et al., 1996; Chalker-Scott, 1999; Ding et al., 2005; Diaz et al., 2006). The effect of low N availability on plant biomass, nitrate uptake, and root architecture has already been widely studied, and it is known that plants modify their root architecture, changing their lateral/primary root ratio and simultaneously decreasing shoot/root ratio in order to forage soil nutrients (Brouwer, 1962; Drew and Saker, 1975; Van der Werf and Nagel, 1996; Lea and Azevedo, 2007; Lemaître et al., 2008).

N use efficiency (NUE) is generally defined as the yield of grain achieved per unit of N available to the crop from soil and applied fertilizer, and it can be divided into two biological components, N uptake efficiency (NUpE) and N utilization efficiency (NUtE) (Moll et al., 1982). Wheat in Northern Europe is typically grown at high N input levels, and N recovery and NUE have been estimated to be between 30 and 65% and 25 kg of DM kg-1 N, respectively (Raun et al., 2002; Sylvester-Bradley and Kindred, 2009; Gaju et al., 2011). A major challenge is optimizing N use (e.g., selecting cultivars that make the best use of applied N; termed here as N-efficient germplasm) while maintaining yield in order to minimize negative environmental impacts and production input costs (Kichey et al., 2007; Kant et al., 2011). A recent study showed that modern cultivars have improved yield performance along with enhanced nutrient use efficiency (Voss-Fels et al., 2019), demonstrating that high NUE is likely to be indirectly increased through selection for high yield.

A prerequisite for breeding and selection of N-efficient germplasm is access to genetic variation. Previous work in wheat field trials conducted under a range of N levels has shown that significant genetic variability exists for NUE along with the component traits (NUpE and NUtE) (Cox et al., 1985; Van Sanford and MacKown, 1986; Dhugga and Waines, 1989; Ortiz-Monasterio et al., 1997; Foulkes et al., 1998; Le Gouis et al., 2000; Brancourt-Hulmel et al., 2003; Barraclough et al., 2010; Gaju et al., 2011, 2014; Cormier et al., 2013; Guttieri et al., 2017; Nehe et al., 2018).

However, the magnitude and relative contribution of the components to overall genetic variability in NUE varies between experiments. For example, NUpE has been shown to account for a greater proportion of variation in NUE at low compared to high N levels (Ortiz-Monasterio et al., 1997; Le Gouis et al., 2000) as has NUtE (Gaju et al., 2011), although Dhugga and Waines (1989) found NUpE to be equally important at both levels.

Breeding and cultivar registration trials are typically conducted at high N levels to ensure maximum expression of genetic potential (Hitz et al., 2016). Voss-Fels et al. (2019) reported that this has also resulted in indirect selection for optimal performance under reduced input scenarios. However, trait heritability within production systems and the magnitude of genotype × production system interaction are key factors when comparing breeding strategies, i.e., direct or indirect selection between conventional (high-input) and organic (or low-input) farming systems (Annicchiarico et al., 2010).

Estimates of heritability at high- and low-input levels together with genetic correlation between input levels have been used for the prediction of relative efficiency of direct vs. indirect selection in wheat to give recommendations for selection programs, aimed at producing cultivars for low-input or organic agriculture (Brancourt-Hulmel et al., 2005; Przystalski et al., 2008; Annicchiarico et al., 2010; Cormier et al., 2013; Šarčević et al., 2014).

Brancourt-Hulmel et al. (2005) and Cormier et al. (2013) concluded that breeding programs aiming to produce N-efficient cultivars for low-input environments should include testing and selection at low input to maximize selection gains for grain yield (GY). Przystalski et al. (2008) suggested combining information from both organic (low input) and non-organic (high input) experiments to optimize the selection of wheat cultivars for organic farming systems, and Hitz et al. (2016) indicated that selection at low N is necessary to identify high NUE genotypes. However, Annicchiarico et al. (2010) found no advantage when targeting organic production of direct selection for GY in organic systems relative to indirect selection in conventional systems. Similarly, Šarčević et al. (2014) reported high efficiency of indirect selection under high N for performance under low N, which was close to 1.0 for GY and for most studied bread-making quality traits.

N use efficiency and its components have been widely studied including in released CIMMYT wheat cultivars (Ortiz-Monasterio et al., 1997), Northwestern European (Le Gouis et al., 2000; Barraclough et al., 2010; Gaju et al., 2011, 2014; Cormier et al., 2013), North American (Guttieri et al., 2017; Russell et al., 2017), and Indian wheats (Nehe et al., 2018). However, the information on variation for these traits is lacking for wheat germplasm adapted to and selected within South-eastern European production conditions.

The objectives of this study were to (1) evaluate the effect of N treatment (low vs. high) on the agronomic performance of a set of wheat cultivars predominantly originating from South-eastern Europe; (2) to estimate variance components and heritability for GY, NUE and its components under low and high N, and related changes caused by breeding and selection; and (3) to identify the relationship between NUE and its components in the cultivar panel to inform future selection and breeding for N efficiency.

## Materials and Methods

### Plant Materials and Field Experiments

Forty-eight winter wheat cultivars were evaluated in field trials under two N fertilization levels over two consecutive seasons (2016/2017 and 2017/2018) in three locations (Osijek, Zagreb, and Poreč) representing different agro-ecological conditions and soil types in Croatia. On average, in both seasons, Osijek was the driest and coolest location, whereas Poreč was the warmest and with the highest amounts of rainfalls (Supplementary File 1_Meteo data 2016–2018).

The panel included current and historical cultivars released between 1936 and 2016 from Croatian breeding institutes (33 cultivars) along with cultivars from breeding programs in seven other countries. The panel was assembled to represent the historical and current significance of cultivars in production in Croatia and their pedigree contributions. Historical cultivars included in the panel were: U-1, San Pastore, and Bezostaya-1 developed in 1936, 1940, and 1959, respectively. The cultivar list, year of registration and their country of origin, and breeding institution are listed in Supplementary Table 1.

Each of the six field trials was set up in a split-plot factorial design with three replicates with N fertilization levels [low N (LN); high N (HN)] as main plots and 48 wheat cultivars as subplots. The harvested plot size was 7.56 m^2^ at Osijek and Poreč and 4.95 m^2^ at Zagreb. Seeding rate was 350 kernels m^–2^ in all trials and for all cultivars. Buffer plots were planted between the main N treatment plots.

The N-min analysis method (Krom, 1980) was used to estimate residual N content in the soil by sampling at two soil depths (30 and 60 cm) before planting. Basic fertilization of 74 kg N ha^–1^, 80 kg P_2_O_5_ ha^–1^, and 120 kg K_2_O ha^–1^ was applied by adding 100 kg ha^–1^ of urea (46% N) and 400 kg ha^–1^ NPK (7:20:30). The N treatment comprised of two N fertilization levels, 0 kg N ha^–1^ (LN) and 100 kg N ha^–1^ (HN), applied as top-dressings of 50 kg N ha^–1^ at tillering (GS23-25 after Zadoks et al., 1974) and stem extension (GS33-35) growth stages, respectively. The HN level (treatment) corresponds to standard N fertilization practice, whereas all other cultural practices, including application of herbicides, insecticides, and fungicides to control major weeds, insects, and foliar diseases, were typical for commercial wheat production in South-eastern Europe.

Descriptions of the soil type, soil N content, and N fertilization rates for HN and LN treatments for the 6-year location combinations are given in Supplementary Table 2.

### Agronomic Trait Measurements and Statistical Analysis

A total of 15 traits were assessed across all trials in order to determine their response to N treatment. All trials were harvested at maturity using plots combine, and GY was adjusted to 0% moisture content (grain dry matter; DM). In order to assess DM, plant material was sampled at flowering (GS63-65) and at harvest maturity (GS92) from middle rows of each experimental plot by cutting samples at the ground level 1 m in length. Fresh samples were weighed, and subsamples of approximately 100 g were dried for 2 days at 70°C to a constant weight and recalculated to 0% moisture basis. Information on dry and fresh weights of whole subsamples and fresh weight of whole samples from the first sampling (GS63-65) was used to calculate the total aboveground dry matter per area at flowering (DMTA_F, kg DM ha^–1^). In subsamples from the second sampling (GS92), spikes were cut at the base and separated from the stems after drying. Spikes were threshed using lab thresher, and chaff collected was returned to the rest of the straw. Harvest index (HI,%) was then calculated as the ratio between grain dry weight and total biomass dry weight of the subsample. The aboveground straw dry matter per area at maturity (DMSA, kg DM ha^–1^) was calculated by dividing GY (kg DM ha^–1^) with HI. Whole subsamples from flowering and straw subsamples from harvest maturity were further subsampled for milling into fine powder (sieves of < 200 μm) using a grinding mill and were used for determination of aboveground plant N content at flowering (NT_F) and straw N content at harvest maturity (NS) using the Kjeldahl method (Kjeldahl, 1883). Grain protein content (GPC) was measured at the Agricultural Institute Osijek using an Infratec 1241 Grain Analyzer (FOSS, Denmark) from the grain samples of 0.5 kg collected after the harvest in all experimental plots. Plant height (PH, cm) was measured at harvest maturity (GS92). Detailed trait descriptions and formulae are given in Supplementary Table 3.

All statistical analysis was performed in R (R Development Core Team, 2013). Detailed formulae and variable descriptions are given in Supplementary Table 4. Three linear models were employed as described by Cormier et al. (2013): first, in all field trials (defined as a location-year-N treatment combination), least squares means were calculated with cultivars and replicates as fixed factors. These adjusted means were then used in the subsequent analyses (second and third models) to determine the contribution of main factors and interaction effects where environment was defined as a combination of years and locations (Supplementary Table 4). The estimation of variance components and testing their significance was done using *lme4* package in R software. Calculations for generalized heritability (H) using formula of Cullis et al. (2006), phenotypic (*r*) and genetic (*r*_*g*_) correlations were done using R/*sommer* (Covarrubias-Pazaran, 2016).

Predicted correlated response of a trait under LN with selection based on a trait mean under HN (*CR*_*LN*_) relative to the predicted response to direct selection under LN (*R*_*LN*_) was calculated according to Falconer and Mackay (1996) (Supplementary Table 4). To avoid bias, as proposed by Weber et al. (2012), the estimates of genetic correlations were allowed to exceed the upper limit of 1, whereas they were restricted to ≤ 1 to get reasonable estimates of indirect selection.

Linear regressions between year of cultivar registration and assessed traits were calculated based on adjusted means. Linear regressions between NUE and its components (NUpE and NUtE) and GY and GPC were calculated using best linear unbiased prediction (BLUP) values. These calculations and respective plotting were done using R software (*lm* function and libraries *ggplot2, ggmisc*, and *ggpubr*).

## Results

### Agronomic Traits Vary Significantly Between Low and High N Levels and Between Testing Environments

The majority of trait means showed significant differences between the LN and HN treatments across testing locations and cultivars tested (Table 1). The difference (% of HN) for production traits, including GY, GPC, GNY, and HI, were all significantly higher at HN compared to LN, whereas NUE, NUtE, NUtE_PROT, NUE_PROT, and BPE were reduced at HN. Two derived traits exhibited the greatest relative differences between treatments with NTA having the largest positive (+ 21.61%) and BPE having the largest negative difference (− 19.43%) between HN and LN. Only five traits (PH, NHI, NUpE, NRE, and PANU) did not show any significant differences between LN and HN treatments.

**TABLE 1:** Trait means at low (LN) and high (HN) fertilization levels across six environments and 48 wheat cultivars.

Trait (unit)	N level		Difference^a^
	LN	HN	(% of HN)
GY (kg DM ha^–1^)	5980.8	6657.4	10.16**
PH (cm)	84.9	85.9	1.16^ns^
GPC (%)	11.2	13.0	13.85***
GNY (kg N ha^–1^)	119.6	150.2	20.37**
NTA (kg N ha^–1^)	143.3	182.8	21.61**
HI (% DM)	46.0	47.9	3.97*
NHI (% N)	83.2	82.4	−0.97^ns^
NUE (kg DM kg^–1^N)	33.1	29.3	−12.97**
NUpE (%)	78.0	80.0	2.5^ns^
NUtE (kg DM kg^–1^ N)	42.7	37.0	−15.41**
NUtE_PROT (% protein kg^–1^ N ha^–1^)	0.09	0.08	−12.50*
NUE_PROT (% protein kg^–1^ N ha^–1^)	0.063	0.059	−6.78*
NRE (%)	65.2	68.9	5.37^ns^
BPE (kg DM kg^–1^ N)	104.5	87.5	−19.43**
PANU (kg N ha^–1^)	59.6	59.6	0.0^ns^

*ns: not significant.*

*, **, and ***: significant at the level of probability p < 0.05, p < 0.01, and p < 0.001, respectively.

*^a^Difference calculated as [(trait mean at HN − trait mean at LN)/trait mean at HN] × 100.*

All traits were normally distributed regardless of N level, although the degree of data dispersion varied by trait/N combination (Supplementary Table 5). Trait means estimated for the 48 cultivars varied at each location, having lower values at LN for GY, GPC, GNY, NTA, and HI in all locations (Supplementary Table 6). For PH, NHI, and BPE, the lowest mean values were recorded in Osijek, whereas the highest values were obtained in Zagreb with higher values of PH and NHI recorded at LN compared to HN, as opposed to Osijek and Poreč locations. The highest calculated mean values of BPE were recorded in Poreč, followed by Zagreb and Osijek. Poreč had the highest values for NUtE and NUtE_PROT at both LN and HN followed by Osijek and Zagreb. Mean values of PANU were found to be significantly higher at Osijek compared to Zagreb (1.4- and 1.2-fold for LN and HN, respectively) and Poreč (1.9- and 1.6-fold for LN and HN, respectively). Unlike the two other locations, mean values of PANU in Osijek were higher under LN compared to HN (Supplementary Table 6). The differences between years at specific N treatment were consistent and significant for PH, NUE, NUtE, NRE, and BPE and consistent but not significant for GY, GNY, and NUpE (Supplementary Table 7). Results by individual location and year were presented in Supplementary Table 8.

### Trait Correlations Are Generally Consistent Across N Treatments and Indicate Linked Responses

Positive correlations (*r* ≥ 0.60) between the analyzed traits at LN and HN and based on varietal adjusted means across the six environments were observed for GY with GNY, HI, NHI, NUE, and NUtE, and at LN for NTA (*r* = 0.71) and NUpE (*r* = 0.69). Strong-to-very-strong negative correlations (*r* ≥ − 0.60) were detected between GY and PH, GPC, NUtE_PROT, and NUE_PROT at both LN and HN levels. At both N levels, GPC showed a positive correlation with PH (*r* = 0.38), NUE_PROT (*r* = 0.95), and NUtE_PROT (*r* = 0.47 and 0.56, at LN and HN, respectively) and a negative correlation with HI (*r* = −0.61 and − 0.64), NHI (*r* = −0.39 and − 0.44), NUtE (*r* = −0.93 and − 0.94), and NUE (*r* = −0.74 and −0.70) at LN and HN, respectively (Table 2). These values were under strong environmental influence and varied depending on the year and location. For example, GY was in correlation with GPC varying from *r* = − 0.30 to *r* = −0.66 (Zagreb in 2018 at LN and Osijek in 2018 at HN), GY with NUpE values varied from the lack of correlation to *r* = 0.80 and with NUtE varied from *r* = 0.46 to *r* = 0.74. GPC was in relatively stable and high negative correlation with NUtE varying from *r* = −0.63 to *r* = − 0.93, whereas this relationship with NUpE was not so consistent (Supplementary File_4 Linear correlations). HI was positively correlated with GNY, NHI, NUE, NUtE, and NRE and negatively correlated with PH, GPC, NUtE_PROT, NUE_PROT, and BPE at both LN and HN.

**TABLE 2:** Linear correlations (*r*) between analyzed traits at low (LN) and high (HN) nitrogen levels across environments (*n* = 48 cultivars).

	N level	PH	GPC	GNY	NTA	HI	NHI	NUE	NUpE	NUtE	NUtE_ PROT	NUE_ PROT	NRE	BPE	PANU
GY	LN	–0.62**	−0.74**	0.81**	0.71**	0.77**	0.62**	0.99**	0.69**	0.80**	−0.61**	−0.65**	0.34*	–0.22	0.42**
	HN	–0.67**	−0.70**	0.74**	0.48**	0.75**	0.60**	0.99**	0.49**	0.75**	−0.89**	−0.63**	0.29*	–0.25	0.38**
PH	LN		0.38**	−0.59**	−0.48**	−0.75**	−0.54**	−0.59**	−0.45**	−0.49**	0.25	0.31*	–0.25	0.52**	–0.22
	HN		0.38**	−0.64**	−0.29*	−0.81**	−0.68**	−0.65**	−0.28*	−0.49**	0.50**	0.33*	−0.38**	0.62**	−0.42**
GPC	LN			–0.24	–0.12	−0.61**	−0.39**	−0.74**	–0.11	−0.93**	0.47**	0.95**	−0.29*	–0.23	–0.16
	HN			–0.07	0.19	−0.64**	−0.44**	−0.70**	0.18	−0.94**	0.56**	0.95**	–0.26	–0.28	0.08
GNY	LN				0.93**	0.64**	0.63**	0.80**	0.90**	0.36*	−0.49**	–0.17	0.25	−0.54**	0.48**
	HN				0.83**	0.50**	0.50**	0.73**	0.83**	0.17	−0.72**	–0.01	0.18	−0.64**	0.64**
NTA	LN					0.40**	0.31*	0.71**	0.99**	0.16	−0.46**	–0.06	0.06	−0.53**	0.54**
	HN					0.04	–0.06	0.46**	0.99**	–0.21	−0.62**	0.22	–0.15	−0.57**	0.62**
HI	LN						0.83**	0.75**	0.34*	0.77**	−0.40**	−0.56**	0.45**	−0.40**	0.19
	HN						0.85**	0.75**	0.05	0.78**	−0.56**	−0.58**	0.47**	−0.38**	0.18
NHI	LN							0.60**	0.25	0.64**	−0.33*	−0.32*	0.58**	−0.29*	0.08
	HN							0.59**	–0.05	0.66**	−0.35*	−0.38**	0.62**	–0.23	0.13
NUE	LN								0.70**	0.80**	−0.63**	−0.65**	0.36*	–0.20	0.40**
	HN								0.49**	0.76**	−0.90**	−0.65**	0.29*	–0.25	0.36*
NUpE	LN									0.13	−0.48**	–0.05	0.05	−0.50**	0.54**
	HN									–0.19	−0.65**	0.20	–0.15	−0.58**	0.61**
NUtE	LN										−0.47**	−0.86**	0.46**	0.12	0.11
	HN										−0.55**	−0.89**	0.42**	0.19	–0.08
NUtE_PROT	LN											0.41**	–0.19	0.25	–0.24
	HN											0.51**	–0.09	0.31*	−0.38**
NUE_PROT	LN												–0.19	–0.24	–0.15
	HN												–0.27	−0.29*	0.09
NRE	LN													–0.10	–0.28
	HN													–0.11	−0.34*
BPE	LN														–0.09
	HN														−0.46**

* and **: Significance (df = 46) at the level of probability p < 0.05 and p < 0.01, respectively.

Of the N-specific derived traits, NUE showed positive correlation with NUtE at both N levels (*r* = 0.80 and *r* = 0.76, at LN and HN, respectively) and with NUpE although the correlation was stronger in LN conditions (*r* = 0.79 and *r* = 0.49, at LN and HN, respectively). Weaker positive correlations were found between NUE and NRE, and PANU at LN (*r* = 0.36 and *r* = 0.40, respectively) and HN (*r* = 0.29 and *r* = 0.36, respectively). At both N levels, NUE was negatively correlated with NUtE_PROT (*r* = − 0.63 and *r* = − 0.90) and NUE_PROT (*r* = − 0.65) (Table 2).

**FIGURE 1:**
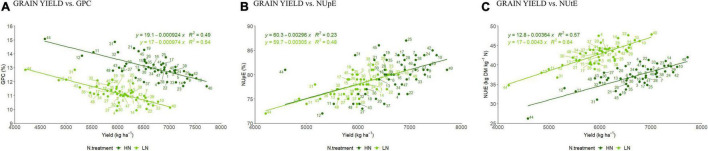
Linear relationship between BLUP values for grain yield (GY in kg ha^–1^) and **(A)** grain protein content (GPC%); **(B)** NUpE and; **(C)** NUtE under LN and HN. Each number represents a discrete cultivar (based on the list in Supplementary Table 1).

Using BLUP means a significant negative correlation was detected between GY and GPC at both N levels (*R*^2^ = 0.54 vs. 0.49 at LN and HN, respectively) (Figure 1A). For the NUE component traits, there was a positive moderate and positive weak relationship with NUpE at LN (*R*^2^ = 0.48) and HN conditions (*R*^2^ = 0.23) (Figure 1B) and moderate positive relationship between GY and NUtE under both LN (*R*^2^ = 0.64) and HN conditions (*R*^2^ = 0.57) (Figure 1C), and positive moderate and positive weak relationship with NUpE at LN (*R*^2^ = 0.48) and HN conditions (*R*^2^ = 0.23) (Figure 1B).

While GY is intrinsically in positive linear relationship with NUE, GPC was in moderate negative relationship at both N levels (*R*^2^ = 0.47 vs. 0.55 at LN and HN, respectively) (Supplementary Figure 1A). Between NUE and its components, a moderate positive relationship was observed for NUE and NUpE under LN (*R*^2^ = 0.49), but a weak relationship was revealed under HN conditions (*R*^2^ = 0.12, Supplementary Figure 1B). NUE was in a strong-to-moderately strong positive relationship with NUtE (*R*^2^ = 0.60 vs. 0.51 at HN and LN, respectively) (Supplementary Figure 1C). NUpE was not linearly significant neither with GPC, nor with NUtE at both LN and HN (Supplementary Figures 2A,B). In contrast, NUtE was in a very strong negative relationship with GPC at both N levels (*R*^2^ = 0.87 vs. 0.89 at LN and HN, respectively; Supplementary Figure 3).

### Decomposition of Phenotypic and Genotypic Variance Shows Genotype-Specific Trait Responses

Breakdown of total phenotypic variation (Table 3) shows significant genotypic and genetic variance effects for all traits, except for NRE. Genotypic effects were largest for PH (69.71%), NUE (42.07%), NUtE (35.67%), and GPC (32.10%). Environmental components of overall variation were highest for BPE (78.54%), NTA (69.11%), GNY (64. 890%), and NRE (63.53%). The G × E interaction was significant for the majority of traits except NUtE_PROT, BPE, and PANU, whereas the G × N interaction was non-significant for all traits. Interaction between N treatment and environment was significant except for NUtE_PROT (Table 3).

**TABLE 3:** Contribution of different sources of variation (%) to total variation of analyzed traits across six environments and two nitrogen levels.

		Sources of variation				
	Genotype (G)	Environment (E)	G × E	G × N	N × E	Residual
GY	19.709***	57.71**	6.39***	0.00^*ns*^	4.21***	11.98
PH	69.71***	16.32***	5.24***	0.00^*ns*^	0.33*	8.40
GPC	32.10***	40.44*	6.72***	0.46^*ns*^	10.97***	9.30
GNY	7.10***	64.89*	3.70***	0.04^*ns*^	13.27***	10.99
NTA	4.85***	69.11**	2.70*	0.19^*ns*^	8.16***	15.0
HI	21.92***	52.76***	6.30***	0.34^*ns*^	1.16*	17.52
NHI	12.64***	36.19^*ns*^	18.56***	0.35^*ns*^	2.75^*ns*^	29.53
NUE	42.07***	3.41^*ns*^	12.21***	0.00^*ns*^	13.97***	28.34
NUpE	18.61***	12.1*	10.3*	0.00^*ns*^	1.54*	57.45
NUtE	35.67***	18.39^*ns*^	5.78***	0.00^*ns*^	15.68***	24.49
NUtE_PROT	5.51**	34.55**	0.14^*ns*^	0.00^*ns*^	0.97^*ns*^	58.82
NUE_PROT	12.76***	66.86***	3.47***	0.00^*ns*^	6.85***	10.06
NRE	0.85^*ns*^	63.53***	3.97***	1.30^*ns*^	11.62***	18.74
BPE	3.59***	78.54***	1.63^*ns*^	0.60^*ns*^	2.54***	13.10
PANU	4.41*	24.61*	6.44^*ns*^	0.00^*ns*^	3.05*	61.49

ns, not significant. *, **, and ***significant variance component at the level of probability p < 0.05, p < 0.01, and p < 0.001, respectively.

### Trait Heritabilities Are Stable Across N Treatments and Performance at High N Predicts Low N Phenotypes

Trait heritabilities across N levels were variable ranging from 0.60 for NUtE_PROT to 0.98 for PH (Table 4) and generally were higher under HN than LN, except for NRE and PANU, but consistent among traits. Of the five directly measured traits, GY, PH, and GPC had high heritability estimates (>0.90), whereas GNY and NTA exhibited considerably lower heritabilities (0.79 and 0.69, respectively). Out of ten derived traits, five traits (HI, NHI, NUE, NUtE, and NUE_PROT) showed higher heritability estimates ranging from 0.88 to 0.95 than the other five traits (NUpE, NUtE_PROT, NRE, BPE, and PANU), whose heritability estimates were in the range from 0.67 to 0.83. Heritabilities at HN and LN were similar for most traits except for NTA and BPE, where a slightly higher heritability was observed at HN and NUtE_PROT, whose heritability was much higher at HN (0.83) than at LN (0.60).

**TABLE 4:** Heritability and genetic correlations (*r*_*g*_) estimates of analyzed traits at two nitrogen levels and across six environments and 48 wheat cultivars and efficiency of indirect selection under HN for performance under LN relative to the predicted response to direct selection under LN (CR_*LN*_/R_*LN*_) for 15 traits.

Trait	Heritability				
	Across N	LN	HN	r_g_ ± s.e.^a^	CR_LN_/R_LN_
GY	0.94 ± 0.01	0.92 ± 0.02	0.92 ± 0.02	1.03 ± 0.02	1.00
PH	0.98 ± 0.00	0.97 ± 0.01	0.98 ± 0.00	1.00 ± 0.0041	1.01
GPC	0.91 ± 0.02	0.95 ± 0.01	0.95 ± 0.01	1.00 ± 0.011	1.00
GNY	0.79 ± 0.07	0.84 ± 0.05	0.88 ± 0.03	1.07 ± 0.054	1.02
NTA	0.69 ± 0.17	0.75 ± 0.01	0.83 ± 0.05	1.12 ± 0.13	1.05
HI	0.95 ± 0.01	0.90 ± 0.02	0.93 ± 0.02	0.99 ± 0.032	1.01
NHI	0.88 ± 0.03	0.84 ± 0.05	0.85 ± 0.04	1.00 ± 0.087	1.01
NUE	0.96 ± 0.01	0.93 ± 0.02	0.94 ± 0.02	1.03 ± 0.023	1.01
NUpE	0.83 ± 0.05	0.81 ± 0.05	0.85 ± 0.06	1.12 ± 0.12	1.02
NUtE	0.91 ± 0.02	0.92 ± 0.02	0.94 ± 0.01	1.00 ± 0.026	1.01
NUtE_PROT	0.75 ± 0.1	0.60 ± 0.45	0.83 ± 0.05	1.21 ± 0.57	1.18
NUE_PROT	0.93 ± 0.02	0.91 ± 0.02	0.92 ± 0.02	0.96 ± 0.041	0.97
NRE	0.67 ± 0.2	0.69 ± 0.17	0.65 ± 0.24	0.42 ± 0.40	0.41
BPE	0.78 ± 0.08	0.77 ± 0.09	0.87 ± 0.03	0.86 ± 0.13	0.91
PANU	0.73 ± 0.12	0.63 ± 0.29	0.62 ± 0.033	1.22 ± 0.61	0.99

*^a^s.e.: standard error of genetic correlation.*

In the present study, genetic correlations between HN and LN were close to 1.0 for GY, PH, and GPC and for derived NUE traits, except for NRE (*r*_*G*_ = 0.42) and BPE (*r*_*G*_ = 0.86). The efficiency of indirect selection under HN for the performance under LN relative to the predicted response to direct selection under LN (CR_*LN*_/R_*LN*_) was near 1.0 for most studied traits, except for NRE (0.41) (Table 4). This indicates that indirect selection under optimal fertilization for these traits will be as efficient as direct selection under reduced fertilization. In the case of NUtE_PROT, indirect selection is expected to be even more efficient than direct selection (CR_*LN*_/R_*LN*_ = 1.18), mainly due to considerably lower heritability under LN than under HN. For NRE and BPE, indirect selection would be less efficient than direct selection.

### Breeding Effects Over Time Have a Significant Impact on Trait Performance at Different Nitrogen Levels

Based on simple linear regression of cultivar trait means on year of registration (as a proxy for breeding progress), the majority of traits showed significant increases over time at both LN and HN (Table 5). A strong positive (increasing) linear relationship was found for GY at both LN and HN (0.31% vs. 0.34% year^–1^), GNY (0.23 vs. 0.25% year^–1^), HI (0.24 vs. 0.28% year^–1^), NHI (for both N levels 0.07% year^–1^), and NUE (0.33 vs. 0.30% year^–1^). A strong decreasing relationship was found for PH (− 0.38 vs.−0. 42% year^–1^) and medium negative relationship with BPE (− 0.13 vs.−0.11% year^–1^). For GPC and PANU, there was inconsistent, significant and non-significant, association with year of release under HN and LN. For NUE_PROT, NUtE_PROT, and NRE, non-significant trends were found (results not shown). Slope of regressions was mostly higher under HN, except for NTA and BPE, and for NHI, which was the same (Table 5).

**TABLE 5:** Linear regression between year of cultivar registration (1936–2016) and analyzed traits^*a*^ at LN and HN levels (N of cultivars = 48).

Trait	b-(Year of registration)	Unit	% Of change per year	*R* ^2^
GY-LN	18.36***	kg DM ha^–1^	0.31	0.32
GY-HN	22.81***		0.34	0.37
PH-LN	−0.321***	cm	–0.38	0.31
PH-HN	−0.358***		–0.42	0.34
GPC-LN	−0.011^*ns*^	%	–0.10	0.07
GPC-HN	−0.013*		–0.10	0.08
GNY-LN	0.277***	kg N ha^–1^	0.23	0.35
GNY-HN	0.382***		0.25	0.33
NTA-LN	0.236***	kg N ha^–1^	0.16	0.26
NTA-HN	0.215*		0.12	0.09
HI-LN	0.111***	%	0.24	0.28
HI-HN	0.133***		0.28	0.33
NHI-LN	0.06***	%	0.07	0.21
NHI-HN	0.06***		0.07	0.21
NUE-LN	0.102***	kg DM kg^–1^ N	0.33	0.35
NUE-HN	0.098***		0.30	0.29
NUpE-LN	0.098***	%	0.15	0.23
NUpE-HN	0.102***		0.12	0.09
NUtE-LN	0.065**	kg DM kg^–1^ N	0.15	0.14
NUtE-HN	0.098**		0.20	0.35
BPE-LN	−0.135*	kg DM kg^–1^ N	-0.13	0.10
BPE-HN	−0.095*		-0.11	0.08
PANU-LN	0.226^*ns*^	kg N ha^–1^	0.38	0.08
PANU-HN	0.301***		0.51	0.25

*^a^Traits where slope coefficients (b) are not statistically significant, at both LN and HN, were not presented.*

*% of change per year—percent change expressed relative to the average trait value.*

***, **, and *: significance of slope coefficients (b) at the level of probability p < 0.001, p < 0.01, and p < 0.05; ns: non-significant.

*R^2^: R-squared value of regression model.*

## Discussion

There are two routes to improving NUE in wheat: improving fertilizer management and/or improving cultivars. The first option is directed at optimizing the application of fertilizer N to crop requirements and weather and soil conditions. The second is to breed cultivars with improved N efficiency in terms of enhanced N uptake and utilization (Barraclough et al., 2010). The importance of these components in relation to NUE will depend on the way of measuring and deriving these traits, the level of N stress, and germplasm diversity used in the evaluation (Han et al., 2015).

In this study, we evaluated the agronomic response of a panel of 48 winter wheat cultivars in six South-eastern European environments at two N levels [representing optimal/standard field N availability (high, HN) and sub-optimal (low, LN)]. Yield and protein significantly decreased by 10% and 14%, respectively, at LN compared to HN. Reductions in grain N yield and aboveground N per unit area were also reduced by approximately 20% at LN. In contrast, the majority of the derived NUE traits had significantly higher mean values at LN compared to HN, ranging from 7% for NUE_PROT to 19% for BPE.

A previous report in Croatian growing conditions showed similar reductions at LN for GY (10%), GPC (13%), and GNY (21%) in a set of 19 European wheat cultivars (Šarčević et al., 2014). Trait responses to reduced N were more pronounced in a study of 225 European wheat cultivars, which showed ∼20% reduction in GY and GPC and a corresponding 30% increase in NUE and NUtE (Cormier et al., 2013). Although the difference in applied N between HN and LN treatments were consistent across studies, the magnitude of trait mean differences suggests the presence of stronger N stress in experiments conducted by Cormier et al. (2013) compared to that of Šarčević et al. (2014) and the present study. Establishment of N stress earlier in the season in the study of Cormier et al. (2013) could be an explanation for the observed differences. These differences could also reflect environmental difference between north-western Europe (Cormier et al., 2013) and assessment in South-eastern European conditions where the wheat growing season is shorter and the more extreme weather conditions lead to lower average yields and a wider gap in yield potential (Schils et al., 2018). In addition to the environmental differences, there is a relationship between yield and N uptake, reflected in the inverse relationship between yield per unit of N uptake and grain protein content (Sadras and Lemaire, 2014), which could explain the differences between studies.

In this study, genotypic (G) variance was significant for all traits, except for NRE, genotype × environment (G × E) variance for all traits, except for NHI, NUtE_PROT, BPE, and PANU, whereas genotype × nitrogen (G × N) interaction was not significant for any trait. Previously, Cormier et al. (2013) reported significant G × N for GY, GPC, and several NUE traits, and significant G × N interactions for GY and other agronomic traits have also been reported in several other wheat studies (Ortiz-Monasterio et al., 1997; Le Gouis et al., 2000; Guarda et al., 2004; Laperche et al., 2006; Barraclough et al., 2010; Gaju et al., 2011). Hitz et al. (2016) suggested that the existence of G × N interaction could be useful for breeders, allowing differentiation of performance between two N environments. In the previous Croatian study by Šarčević et al. (2014), G × N interaction was not significant for GY, but it was significant for all grain quality traits and for most rheological parameters, for which the negative effect of reduced N fertilization was much stronger as compared to its effect on GY. Other recent studies by Guttieri et al. (2017) and Russell et al. (2017) found no significant G × N interactions for GY or any NUE traits for wheat grown in field experiments in the United States. A recent study showed that G × N interactions for GY in wheat were more frequently observed when three or more N rates were used in the experimental design (Brasier et al., 2020), indicating that further experimentation is likely required to robustly dissect G × N interactions.

In the present study, genetic correlations between HN and LN were high for GY, GPC, and for derived NUE traits, reflecting the absence of significant G × N interaction for these traits (Table 4). Heritability estimates for analyzed traits were either similar for the two N fertilization levels or higher at HN than LN. Reflecting this, the efficiency of indirect selection at HN for performance at LN relative to direct selection at LN (indirect selection efficiency, ISE) was ≥ 1 for most traits. Therefore, we conclude that indirect selection for GY, GPC, and most other studied traits under optimal fertilization will be at least as efficient as direct selection under reduced fertilization. This is in agreement with previous assessments of Croatian (Šarčević et al., 2014) and northern European (Voss-Fels et al., 2019) wheat and has major implications for the selection of wheat cultivars for N efficiency. Previous studies have reported lower ISE estimates, although these have been in high N stress comparisons, which exhibit much larger reductions between LN and HN for GY and/or GPC (Brancourt-Hulmel et al., 2005; Cormier et al., 2013). This is in line with the conclusion of Cormier et al. (2016), who found that indirect selection is efficient in moderate N stresses, but it does not surpass direct selection in extreme low N conditions. Similarly, Hitz et al. (2016), studying NUE and related traits in US winter wheats under contrasting N fertilization rates, which resulted in severe N stress, concluded that without screening breeding lines in low N environments concurrently, it will not be possible to identify high NUE genotypes. However, in a European context, it is unlikely that the target environment will be characterized as low N outside of specialist organic production. However, the occurrence of moderate N stress is much more likely within the current production and breeding framework in areas with generally low soil fertility, decreased water availability due to drought or adverse weather conditions or in zones, which have restrictions on N fertilizer use due to run-off concerns. This is also confirmed in the present study, where the effect of location (Poreč vs. two other locations) was more pronounced than the effect of LN treatment in reducing GY (Supplementary Table 6). A similar pattern was observed in the study of Gaju et al. (2011), who reported that in four out of seven environments, mean GY of 16 European wheat cultivars grown at LN was significantly higher than the corresponding GY means at both HN and LN in the lowest yielding environment, although the difference in the amount of N fertilizer applied between HN and LN treatments was as high as 200 kg N ha^–1^. In a later study, Gaju et al. (2014) compared the N accumulation in crop components at anthesis between the highest and lowest yielding location from the same experiment and found significantly higher content of N in leaf lamina, stem-and-leaf-sheath, and ear at the highest yielding location, regardless of the level of N fertilization. Similarly, in the study of Brasier et al. (2020), including 12 US soft red winter wheat lines and cultivars, the effect of year on GY was more pronounced than the effect of N fertilization applied in the range from 45 to 134 kg N ha^–1^.

In practical terms, promising breeding material is trialed across multiple environments, and it is likely this exposes lines to a mix of HN and moderate LN environments (Cormier et al., 2013). This is likely to explain the similarity in genetic progress at HN and LN seen in this, and previous studies (Voss-Fels et al., 2019). Further optimization of the multi-environment framework could incorporate a reduced N fertilizer rate at a limited number of locations in order to increase the frequency of stress environments.

This has been previously explored as a component of a NUE selection framework by Cormier et al. (2013) and would give better insight into the production yield stability of a genotype. This would be particularly useful for evaluating bread-making quality properties, which have been shown to be more sensitive to reduced N fertilization than GY (Šarčević et al., 2014).

Our analysis shows that significant genetic (breeding) progress has been made for yield and the majority of analyzed traits at both LN and HN in the 48 cultivars assessed. Genetic improvement in GY was estimated at 0.31 and 0.34% year^–1^, with concomitant decreases in PH (− 0.38 and−0. 42% year^–1^) and BPE (−0.13 and −0.11% year^–1^) at LN and HN, respectively. As a consequence of reducing PH and BPE while increasing GY, HI was increased by 0.24 and 0.28% year^–1^ at LN and HN, respectively.

From the perspective of genetic improvement for NUE, Cormier et al. (2013) estimated NUE progress in European wheat between 1985 and 2010 to be 0.37 and 0.30% year^–1^, respectively, at LN and HN. Ortiz-Monasterio et al. (1997) reported genetic progress for NUE at 0.4–1.1% year^–1^ depending on the N levels in CIMMYT’s spring wheat released between 1962 and 1985.

In the present study, NUE progress between 1936 and 2016 was 0.33 and 0.30% year^–1^, which translates to genetic improvement in NUE of 8.26 and 7.94 kg of DM kg^–1^ N or in total 26.7 and 24.3% at LN and HN, respectively.

Similar levels of genetic gain have been recorded in Canadian spring wheat (Kubota et al., 2018; 0.34% year^–1^ in NUE under HN). As NUE is a complex trait and is defined as the product of NUpE and NUtE (Moll et al., 1982), its improvement should be realized through selection on one of its components. In this study, we found that NUtE had a stronger effect on NUE than NUpE based on estimated correlation coefficients (0.80 vs. 0.70 at LN, and 0.76 vs. 0.49 at HN). This is in agreement with other authors who found genetic variation in NUE more related to NUtE (Brancourt-Hulmel et al., 2003; Gaju et al., 2011; Uzik et al., 2012; Cormier et al., 2013). However, several other studies have reported similar contributions (Van Sanford and MacKown, 1986) or dominance of NUpE (Dhugga and Waines, 1989), particularly at LN (Ortiz-Monasterio et al., 1997; Le Gouis et al., 2000). We found both positive impacts of breeding on both NUpE (an annual increase of 0.15 and 0.12%), and for NUtE (0.15 and 0.20% at LN and HN, respectively).

As in other reports (e.g., Voss-Fels et al., 2019), NUE measured under LN was higher than that measured under HN in our dataset (Table 1). This suggests that the cultivars selected thus far have an inherent capacity for greater NUE, which declines under HN conditions. Identifying cultivars that can maintain high NUE in HN conditions should enable yield improvement with a lower environmental impact. An alternative target to NUE has been proposed by Sylvester-Bradley and Kindred (2009), who defined the economic N optima as the N level necessary to achieve a high yield with the lowest input cost to maximize profits. Although the economic N optima cannot be used as a breeding target *per se*, it highlights the relevance of N responsiveness to the improvement of NUE (Swarbreck et al., 2019). Cultivars showing high N responsiveness that is maintained under high N show lower economic N optima. Here, selection under varying N levels evaluates a genotype’s N responsiveness (Sylvester-Bradley et al., 2015), potentially allowing for the selection of cultivars that are highly responsive to both low N and high N conditions. The ideal genotype should possess high genetic N efficiency and high N responsiveness, so traits for N efficiency and responsiveness should not be genetically linked in order to select genotype that performs better under both conditions (Han et al., 2015). For producers, on the other side, De Oliveira Silva et al. (2020) found that genotypes with high mean response and high variability in their response to higher N levels across years could offer greater opportunities to maximize yield.

In summary, we report that while agronomic and N-related traits vary significantly between N levels and environments, traits are consistently correlated irrespective of N level. All of the cultivars tested showed a common directional response at LN and HN, and performance at HN generally predicted performance at LN although direct selection offers opportunities to optimize some N-related traits. Analysis of breeding progress revealed improvements in NUE over time linked to yield improvement. Our results are significant for informing future selection and breeding for N efficiency in South-eastern European wheat and add further evidence confirming that selection at optimal N is most relevant for delivering enhanced N efficiency in wheat.

## Data Availability Statement

The original contributions presented in the study are included in the article/Supplementary Material, further inquiries can be directed to the corresponding author/s.

## Author Contributions

DN, HŠ, KD, BP, ZL, and AB contributed to the conceptualization of the study. DN, HŠ, SG, KD, MI, BP, ZL, and IP contributed to the methodology. MI, DN, IP, and SS conducted the formal analysis. MI, BR, MM, AL, and MČ performed the investigations. MI and IP performed data curation. MI, IP, SG, KD, DN, HŠ, AL, and MČ involved in writing the original draft preparation. AB, SS, BP, ZL, and KD contributed to writing, reviewing, and editing. DN, HŠ, AB, and SS supervised the work. All authors contributed to manuscript revision, read, and approved the submitted version.

## Conflict of Interest

The authors declare that the research was conducted in the absence of any commercial or financial relationships that could be construed as a potential conflict of interest.

## Publisher’s Note

All claims expressed in this article are solely those of the authors and do not necessarily represent those of their affiliated organizations, or those of the publisher, the editors and the reviewers. Any product that may be evaluated in this article, or claim that may be made by its manufacturer, is not guaranteed or endorsed by the publisher.

## Supplementary Material

Supplementary File 1 (Meteo data 2016–2018) | The meteorological data file contains measurements taken at three weather stations of experimental sites (Osijek, Poreč, and Zagreb) during three consecutive years (2016, 2017 and 2018), and includes the information on average daily air temperatures (Sheet1), precipitations (Sheet 2), and summary values (Sheet 3).
