# Supplementary material for: Variation for Nitrogen Use Efficiency Traits in Wheat Under Contrasting Nitrogen Treatments in South-Eastern Europe
Source: Front Plant Sci. 2021 Nov 18;12:682333. doi: 10.3389/fpls.2021.682333 (PMC8636685; doi:10.3389/fpls.2021.682333)
Supplement: Supplementary File 1 — (Meteo data 2016–2018) | The meteorological data file contains measurements taken at three weather stations of experimental sites (Osijek, Poreč, and Zagreb) during three consecutive years (2016, 2017 and 2018), and includes the information on average daily air temperatures (Sheet1), precipitations (Sheet 2), and summary values (Sheet 3). [file Data_Sheet_1.zip › Supplementary File 2_ Supplementary Tables 1-8.docx]

**Supplementary Table 1. List of wheat cultivars, year of registration, country of origin and breeding institution.**

| **ID** | **Cultivar** | **Year of registration** | **Country*/ Breeding institution** |
| --- | --- | --- | --- |
| 1 | ANDJELKA | 2008 | HR / Agricultural Institute Osijek |
| 2 | BC ANICA | 2012 | HR / BC Institute Zagreb |
| 3 | BC BERNARDA | 2015 | HR / BC Institute Zagreb |
| 4 | BC CERTISSA | 2014 | HR / BC Institute Zagreb |
| 5 | BC DARIJA | 2012 | HR / BC Institute Zagreb |
| 6 | BC IRENA | 2010 | HR / BC Institute Zagreb |
| 7 | BC IRMA | 2015 | HR / BC Institute Zagreb |
| 8 | BC LORENA | 2012 | HR / BC Institute Zagreb |
| 9 | BC LJEPOTICA | 2016 | HR / BC Institute Zagreb |
| 10 | BC OPSESIJA | 2016 | HR / BC Institute Zagreb |
| 11 | BC TENA | 2011 | HR / BC Institute Zagreb |
| 12 | BEZOSTAYA-1 | 1959 | RU/KRIA P.P. Lukyanenko |
| 13 | BOLOGNA | 2001 | FR/ETS CC BENOIST |
| 14 | CALISOL | 2012 | FR/SARL Adrien Momont et Fils  (FR) |
| 15 | DROPIA | 1993 | RO/INCDA Fundulea |
| 16 | FELIX | 2007 | HR / Agricultural Institute Osijek |
| 17 | FICKO | 2007 | HR / Agricultural Institute Osijek |
| 18 | FLAMURA 85 | 2011 | RO/INCDA Fundulea |
| 19 | FORCALI | 2013 | FR/SARL Adrien Momont et Fils  (FR) |
| 20 | GALLOPER | 2015 | HR / Agricultural Institute Osijek |
| 21 | GOLUBICA | 1998 | HR / Agricultural Institute Osijek |
| 22 | GRAINDOR | 2006 | FR/Unisigma GIE (FR) |
| 23 | ISENGRAIN | 1997 | FR/Florimond Desprez Veuve et Fils (FR) |
| 24 | KATARINA | 2006 | HR / Agricultural Institute Osijek |
| 25 | KRALJICA | 2010 | HR / Agricultural Institute Osijek |
| 26 | LUKULLUS | 2008 | AT/Saatzucht Donau Ges.m.b.H. & CoKG |
| 27 | MIHELCA | 1996 | HR / BC Institute Zagreb |
| 28 | MV-NADOR | 2014 | HU/MTA Agrartudomanyi Kutatokoezpont |
| 29 | MV-NEMERE | 2013 | HU/MTA Agrartudomanyi Kutatokoezpont |
| 30 | OSJEČKA CRVENKA | 1976 | HR / Agricultural Institute Osijek |
| 31 | OS-JELENA | 2014 | HR / Agricultural Institute Osijek |
| 32 | OS-OLIMPIJA | 2009 | HR / Agricultural Institute Osijek |
| 33 | PRIMA | 2001 | HR / BC Institute Zagreb |
| 34 | REBEKA | 2011 | HR / Agricultural Institute Osijek |
| 35 | RENAN | 1990 | FR/Institut National de la Recherche Agronomique |
| 36 | SAN PASTORE | 1940 | IT/[Istituto Nazionale di Genetica per la Cerealicoltura Ce](https://it.wikipedia.org/wiki/Istituto_Nazionale_di_Genetica_per_la_Cerealicoltura)realicoltura |
| 37 | SANA | 1983 | HR / BC Institute Zagreb |
| 38 | SILVIJA | 2010 | HR / Agricultural Institute Osijek |
| 39 | SLAVONIJA | 1984 | HR / Agricultural Institute Osijek |
| 40 | SOFRU | 2013 | FR/Caussade Semences S.A. |
| 41 | SRPANJKA | 1989 | HR / Agricultural Institute Osijek |
| 42 | TIKA-TAKA | 2014 | HR / Agricultural Institute Osijek |
| 43 | TOSUNBEY | 2004 | TR/Field Crops Central Institute |
| 44 | U-1 | 1936 | HR / Agricultural Institute Osijek |
| 45 | VIKTORIA | 2011 | HR/Agrigenetics d.o.o. |
| 46 | VULKAN | 2009 | HR / Agricultural Institute Osijek |
| 47 | WALDINGER | 2016 | HR / Agricultural Institute Osijek |
| 48 | ZLATNA DOLINA | 1971 | HR / BC Institute Zagreb |

*HR-Croatia, HU-Hungary, FR-France, RU- Russia, RO-Romania, AT- Austria, IT-Italy, TR-Turkey

**Supplementary Table 2. Soil N content (kg ha^-1^) at three locations (Osijek, Poreč and Zagreb) in 2016/17 and 2017/18.**

**NTAmax corresponds to the 95th percentile of total nitrogen per area at maturity (GS 92) for all the cultivars present in the trial and is an estimate of N available (soil + fertilizer N)**

| **Location** | **Soil type** | **Season** | **Residual soil N**  **(kg N ha^-1^)** | **Basic N fertilization**  **(kg N ha^-1^)** | **N top-dressing**  **(kg N ha^-1^)** | | **Total N**  **(kg N ha^-1^)** | | **NTAmax**  **(kg N ha^-1^)** | |
| --- | --- | --- | --- | --- | --- | --- | --- | --- | --- | --- |
|  |  |  |  |  | LN | HN | LN | HN | LN | HN |
| Osijek | Eutric  cambisol | 2016/17 | 20 | 74 | 0 | 50+50 | 94 | 194 | 225 | 262 |
|  |  | 2017/18 | 42 | 74 | 0 | 50+50 | 116 | 216 | 168 | 226 |
| Poreč | Red soil | 2016/17 | 14 | 74 | 0 | 50+50 | 88 | 188 | 122 | 158 |
|  |  | 2017/18 | 44 | 74 | 0 | 50+50 | 118 | 218 | 159 | 234 |
| Zagreb | Carbonate  alluvium | 2016/17 | 27 | 74 | 0 | 50+50 | 101 | 201 | 201 | 235 |
|  |  | 2017/18 | 38 | 74 | 0 | 50+50 | 112 | 212 | 221 | 249 |

**Supplementary Table 3. Trait description.**

| **Trait** | **Unit** | **Description** | **Formula** |
| --- | --- | --- | --- |
| DMTA_F | kg DM ha^-1^ | Total aboveground dry matter per area at flowering |  |
| NT_F | % | Aboveground plant N content at flowering |  |
| DMSA | kg DM ha^-1^ | Above-ground straw dry matter per area at maturity |  |
| NS | % | Straw N content at maturity |  |
| NTA_F | kg N ha^-1^ | Total aboveground N per area at flowering | DMTA_F × (NT_F / 100) |
| NSA | kg N ha^-1^ | Straw N per area at maturity | DMSA × (NS / 100) |
| GY | kg DM ha^-1^ | Grain yield (grain dry matter) per area |  |
| PH | cm | Plant height |  |
| GPC | % | Grain protein content |  |
| GNY | kg N ha^-1^ | Grain N yield | [GPC / (5.7 × 100)] × GY |
| NTA | kg N ha^-1^ | Total aboveground N per area at maturity | GNY + NSA |
| NTAmax | kg N ha^-1^ | Maximal N per area | 95th percentile of NTA per trial |
| HI | % | Harvest index | [GY / (GY + DMSA)] × 100 |
| NHI | % | N harvest index | (GNY / NTA) × 100 |
| NUE | kg DM kg^-1^ N | N use efficiency | GY / NTAmax |
| NUpE | % | N uptake efficiency at maturity | (NTA / NTAmax) × 100 |
| NUtE | kg DM kg^-1^ N | N utilization efficiency | GY / NTA |
| NUtE_PROT | % protein kg^-1^ N ha^-1^ | N utilization efficiency to protein | GPC/NTA |
| NUE_PROT | % protein kg^-1^ N ha^-1^ | N use efficiency to protein | GPC/NTAmax |
| NRE | % | N remobilization efficiency | [(NTA_F - NSA) / NTA_F] × 100 |
| BPE | kg DM kg^-1^ N | Biomass production efficiency | (GY + DMSA) / NTA |
| PANU | kg N ha^-1^ | Post-anthesis N uptake | NTA - NTA_F |

**Supplementary Table 4. Formulae and references used in calculations.**

| **ID formulae** | **Formula description and reference** | **Formulae** |
| --- | --- | --- |
| 1 | First linear model  **Cormier et al. (2013)** | $Y_{ij}= \mu+REP+{GEN}_{i}+\varepsilon$  $Y_{ij}$is the phenotype of genotype *i* in single trial (location-year-N treatment combination) analysis  μ is the general mean  $\text{REP}$ is the fixed effect of replicate  ${GEN}_{i}$ is the fixed effect of genotype *i*  $\varepsilon$is residual error term |
| 2 | Second linear model  **Cormier et al. (2013** | $Y_{ijl} = \mu+N_{l}+{GEN}_{i}+{ENV}_{j}+N_{l}{\times GEN}_{i}+N_{l}{\times ENV}_{j}+{GEN}_{i}{\times ENV}_{j}+\varepsilon_{ijl}$  $Y_{ijl}$ is the phenotype of genotype *i* in environment *j* at N treatment *l* (0 kg/ha and 100 kg/ha of N applied in top-dressing)  $\mu$ is the general mean  $N_{l}$is the fixed effect of N treatment *l*  ${GEN}_{i}$ is random additive effect of genotype *i*  ${ENV}_{j}$ is the random effect of environment *j* (location-year combination)  $N_{l}{\times GEN}_{i}$ ; $N_{l}{\times ENV}_{j}$ ; ${GEN}_{i}{\times ENV}_{j}$ are random interaction terms  $\varepsilon_{ijl}$ is residual error term |
| 3 | Third linear model  **Cormier et al. (2013)** | $Y_{ij}= \mu+{GEN}_{i}+{ENV}_{j}+\varepsilon_{ij}$  $Y_{ij}$is the phenotype of genotype *i* in environment *j* in single N treatment analysis across environments  $\mu$ is the general mean  ${GEN}_{i}$ is random additive effect of genotype *i*  ${ENV}_{j}$ is the random effect of environment *j* (location-year combination)  $\varepsilon_{ij}$is residual error term |
| 4 | Generalized heritability  **Cullis et al. (2006)** | $H=1-PEV/(2\times\sigma_{g}^{2})$  $H$ is generalized heritability  $PEV$ is average pairwise prediction error variance  $\sigma_{g}^{2}$ is random genetic variance |
| 5 | Genetic correlations between individual traits at two N levels  **Falconer and Mackay (1996)** | $r_{g}={Cov}_{trait LN:HN}/sqrt (\sigma_{g-trait LN}^{2}\times\sigma_{g-trait HN}^{2})$  ${Cov}_{trait LN:HN}$ is trait genetic covariance at two N levels  $\sigma_{g-trait LN}^{2}$ is trait genetic variance at LN level  $\sigma_{g-trait HN}^{2}$ is trait genetic variance at HN level |
| 6 | Predicted correlated response of a trait under LN with selection based on a trait mean under HN  **Falconer and Mackay (1996)** | ${CR}_{LN}/R_{LN}= r_{g}\times{(H_{HN}/H_{LN})}^{1/2}$ |

**Supplementary Table 5. Descriptive statistics for analyzed traits.**

| **Trait** | **Unit** | **N level** | **Mean** | **Min** | **Max** | **Std. Dev. (±)** |
| --- | --- | --- | --- | --- | --- | --- |
| GY | kg DM ha^-1^ | LN | 5980.8 | 2432.3 | 9330.2 | 1249.1 |
|  |  | HN | 6657.4 | 2683.0 | 9782.5 | 1328.8 |
|  |  | Average | 6319.1 | 2432.3 | 9782.5 | 1332.2 |
| PH | cm | LN | 84.9 | 61.3 | 149.3 | 12.71 |
|  |  | HN | 85.9 | 62.7 | 154.7 | 12.84 |
|  |  | Average | 85.4 | 61.3 | 154.7 | 12.77 |
| GPC | % | LN | 11.2 | 7.7 | 15.2 | 1.33 |
|  |  | HN | 13.0 | 10.2 | 16.8 | 1.24 |
|  |  | Average | 12.1 | 7.7 | 16.8 | 1.57 |
| GNY | kg N ha^-1^ | LN | 119.6 | 46.1 | 182.0 | 30.7 |
|  |  | HN | 150.2 | 65.8 | 209.9 | 33.3 |
|  |  | Average | 134.9 | 46.1 | 209.9 | 35.5 |
| NTA | kg N ha^-1^ | LN | 143.3 | 58.1 | 214.3 | 34.9 |
|  |  | HN | 182.8 | 82.6 | 256.2 | 38.1 |
|  |  | Average | 163.1 | 58.1 | 256.2 | 41.5 |
| HI | % | LN | 46.0 | 24.8 | 64.7 | 7.5 |
|  |  | HN | 47.9 | 17.7 | 64.6 | 7.8 |
|  |  | Average | 46.9 | 17.7 | 64.7 | 7.7 |
| NHI | % | LN | 83.2 | 64.8 | 93.9 | 5.2 |
|  |  | HN | 82.4 | 42.9 | 93.1 | 6.7 |
|  |  | Average | 82.8 | 42.9 | 93.9 | 6.0 |
| NUE | kg DM kg^-1^ N | LN | 33.1 | 14.5 | 42.3 | 4.3 |
|  |  | HN | 29.3 | 12.5 | 41.9 | 4.0 |
|  |  | Average | 31.2 | 12.5 | 42.3 | 4.4 |
| NUpE | % | LN | 78.1 | 47.0 | 100.0 | 9.0 |
|  |  | HN | 80.0 | 47.0 | 100.0 | 10.1 |
|  |  | Average | 79.2 | 47.0 | 100.0 | 9.5 |
| NUtE | kg DM kg^-1^ N | LN | 42.7 | 26.5 | 59.7 | 5.4 |
|  |  | HN | 37.0 | 15.1 | 47.6 | 4.3 |
|  |  | Average | 39.9 | 15.1 | 59.7 | 5.7 |
| NUtE PROT | % protein kg^-1^ N ha^-1^ | LN | 0.09 | 0.05 | 0.50 | 0.03 |
|  |  | HN | 0.08 | 0.05 | 0.21 | 0.02 |
|  |  | Average | 0.08 | 0.05 | 0.50 | 0.03 |
| NUE_PROT | % protein kg^-1^ N ha^-1^ | LN | 0.063 | 0.04 | 0.09 | 0.01 |
|  |  | HN | 0.059 | 0.04 | 0.07 | 0.01 |
|  |  | Average | 0.06 | 0.04 | 0.09 | 0.01 |
| NRE | % | LN | 65.2 | 22.7 | 95.0 | 17.6 |
|  |  | HN | 68.9 | 16.3 | 96.0 | 17.9 |
|  |  | Average | 67.1 | 16.3 | 96.0 | 17.8 |
| BPE | kg DM kg^-1^ N | LN | 104.5 | 64.6 | 224.8 | 27.8 |
|  |  | HN | 87.5 | 52.2 | 162.2 | 22.5 |
|  |  | Average | 96.0 | 52.2 | 224.8 | 26.7 |
| PANU | kg N ha^-1^ | LN | 59.6 | 5.7 | 378.3 | 37.1 |
|  |  | HN | 59.6 | 8.9 | 181.4 | 27.9 |
|  |  | Average | 59.6 | 5.7 | 378.3 | 32.8 |

**Supplementary Table 6. Trait means (standard deviation(±))across 48 wheat cultivars and two years in low N (LN) and high N (HN) treatments at three locations (Osijek, Poreč and Zagreb).**

| **Trait** | **Osijek** | | **Poreč** | | **Zagreb** | |
| --- | --- | --- | --- | --- | --- | --- |
|  | **LN** | **HN** | **LN** | **HN** | **LN** | **HN** |
| **GY** | 6239.1 (1329.4) | 6988.5 (1165.3) | 5053.3 (936.7) | 5694.1 (1288.5) | 6650.1 (824.5) | 7289.8 (929.1) |
| **PH** | 80.5 (13.5) | 82.7 (13.6) | 86.0 (12.7) | 86.8 (13.4) | 88.4 (10.5) | 88.3 (10.9) |
| **GPC** | 11.4 (0.9) | 13.3 (1.0) | 10.1 (1.1) | 12.5 (1.3) | 12.1 (1.1) | 13.2 (1.2) |
| **GNY** | 123.5 (23.8) | 161.4 (23.2) | 90.2 (22.0) | 126.6 (36.4) | 145.1 (16.0) | 162.7 (25.5) |
| **NTA** | 150.6 (28.8) | 195.3 (27.7) | 108.8 (22.6) | 152.4 (42.4) | 170.5 (18.2) | 200.7 (20.3) |
| **HI** | 48.3 (6.1) | 49.7 (6.2) | 41.1 (7.0) | 43.6 (7.1) | 48.6 (6.6) | 50.3 (8.1) |
| **NHI** | 82.2 (3.7) | 83.0 (4.3) | 82.3 (5.9) | 83.1 (4.8) | 85.2 (5.3) | 81.2 (9.6) |
| **NUE** | 31.7 (4.5) | 28.7 (4.3) | 35.9 (4.5) | 29.1 (3.9) | 31.6 (4.3) | 30.2 (4.2) |
| **NUpE** | 77.0 (9.0) | 80.0 (10.0) | 77.0 (9.0) | 77.0 (10.0) | 81.0 (8.0) | 83.0 (9.0) |
| **NUtE** | 41.6 (3.9) | 36.0 (3.6) | 47.1 (4.8) | 38.3 (4.5) | 39.4 (4.3) | 36.8 (4.5) |
| **NUtE_PROT** | 0.08 (0.03) | 0.07 (0.01) | 0.10 (0.04) | 0.09 (0.03) | 0.07 (0.01) | 0.07 (0.01) |
| **NUE_PROT** | 0.06 (0.01) | 0.05 (0.01) | 0.07 (0.01) | 0.07 (0.01) | 0.06 (0.01) | 0.05 (0.01) |
| **NRE** | 60.9 (12.5) | 70.1 (9.5) | 67.2 (21.3) | 73.6 (14.8) | 67.5 (17.2) | 62.9 (24.5) |
| **BPE** | 88.7 (12.2) | 76.3 (11.7) | 125.8 (33.0) | 100.5 (27.8) | 99.1 (18.9) | 85.60 (17.9) |
| **PANU** | 79.3 (41.6) | 73.1 (27.3) | 41.5 (35.1) | 45.7 (24.0) | 58.0 (21.9) | 59.9 (25.7) |

**Supplementary Table 7. Trait means (standard deviation(±)) across 48 cultivars and three locations in low N (LN) and high N (HN) treatments in two testing seasons (2016/17 and 2017/18).**

| **Trait** | **LN** | |  | **HN** | |  |
| --- | --- | --- | --- | --- | --- | --- |
|  | **2017** | **2018** | **p** | **2017** | **2018** | **p** |
| **GY** | 6088.4 (1498.9) | 5873.2 (928.2) | 0.144 | 6541.2 (1659.6) | 6773.7 (872.9) | 0.138 |
| **PH** | 86.9 (11.9) | 83.0 (13.2) | 0.008 | 88.2 (13.1) | 83.6 (12.2) | 0.002 |
| **GPC** | 11.0 (1.6) | 11.4 (0.9) | 0.013 | 12.8 (1.3) | 13.2 (1.1) | 0.004 |
| **GNY** | 119.4 (37.5) | 119.8 (22.1) | 0.906 | 147.9 (43.5) | 152.5 (18.1) | 0.247 |
| **NTA** | 142.2(40.7) | 144.5 (28.1) | 0.580 | 174.7 (48.1) | 190.9 (21.6) | <0.001 |
| **HI** | 44.9 (7.7) | 47.1 (7.0) | 0.011 | 47.5 (8.3) | 48.2 (7.2) | 0.427 |
| **NHI** | 83.1 (5.5) | 83.4 (4.9) | 0.692 | 84.3 (5.2) | 80.6 (7.6) | <0.001 |
| **NUE** | 33.7 (4.8) | 32.46 (4.8) | 0.029 | 30.0 (4.6) | 28.7 (3.6) | 0.008 |
| **NUpE** | 0.77 (0.09) | 0.79 (0.09) | 0.114 | 0.79 (0.11) | 0.81 (0.09) | 0.212 |
| **NUtE** | 43.9 (5.4) | 41.5 (5.3) | <0.001 | 38.0 (4.1) | 36.0 (4.3) | <0.001 |
| **NUtE_PROT** | 0.09 (0.04) | 0.08 (0.02) | 0.187 | 0.08 (0.03) | 0.07 (0.01) | <0.001 |
| **NUE_PROT** | 0.06 (0.01) | 0.06 (0.01) | 0.668 | 0.06 (0.01) | 0.06 (0.01) | <0.001 |
| **NRE** | 63.1 (17.7) | 67.4 (17.2) | 0.037 | 72.0 (13.1) | 65.8 (21.4) | 0.003 |
| **BPE** | 110.5 (32.8) | 98.5 (20.0) | <0.001 | 93.5 (25.7) | 81.5 (16.9) | <0.001 |
| **PANU** | 60.9 (28.1) | 58.2 (44.4) | 0.539 | 54.1 (25.1) | 65.0 (29.6) | 0.001 |

p-significance level of differences between years at specific nitrogen treatment

**Supplementary Table 8. Trait means (standard deviation(±)) across 48 cultivars at three locations under low N (LN) and high N (HN) treatments in two testing seasons (2016/17 and 2017/18).**

| **Trait** |  | | **LN** |  | |  |  |  | **HN** |  |  |  |
| --- | --- | --- | --- | --- | --- | --- | --- | --- | --- | --- | --- | --- |
|  |  | **2017** |  |  | **2018** |  |  | **2017** |  |  | **2018** |  |
|  | **OSIJEK** | **POREČ** | **ZAGREB** | **OSIJEK** | **POREČ** | **ZAGREB** | **OSIJEK** | **POREČ** | **ZAGREB** | **OSIJEK** | **POREČ** | **ZAGREB** |
| **GY** | 7241.3 (954.6) | 4344.2 (666.2) | 6679.7 (812.0) | 5236.9 (780.8) | 5762.3 (550.3) | 6620.4 (844.2) | 7502.4 (1128.9) | 4618.1 (772.6) | 7503.1 (918.3) | 6474.5 (964.8) | 6770.0 (628.0) | 7076.5 (899.1) |
| **PH** | 87.0 (14.0) | 87.1 (13.3) | 86.7 (7.5) | 74.0 (9.3) | 84.9 (12.1) | 90.1 (12.7) | 89.5 (13.9) | 87.4 (14.4) | 87.7 (10.9) | 75.8 (9.2) | 86.1 (12.4) | 88.8 (10.9) |
| **GPC** | 11.2 (0.9) | 9.2 (0.7) | 12.5 (1.0) | 11.5 (0.8) | 10.9 (0.8) | 11.7 (1.0) | 13.0 (0.9) | 11.5 (0.9) | 13.8 (1.0) | 13.5 (1.1) | 13.5 (0.8) | 12.6 (1.2) |
| **GNY** | 141.9 (16.6) | 70.2 (9.5) | 145.9 (14.1) | 105.0 (13.2) | 110.1 (8.3) | 144.2 (17.8) | 170.8 (23.8) | 93.0 (13.8) | 180.0 (18.1) | 151.9 (18.4) | 160.3 (13.2) | 145.3 (19.3) |
| **NTA** | 173.3 (19.3) | 89.6 (12.2) | 163.7 (14.9) | 128.0 (16.0) | 128.0 (11.3) | 177.4 (18.7) | 208.2 (27.3) | 114.2 (16.8) | 201.7 (20.0) | 182.4 (21.5) | 190.6 (19.4) | 199.6 (20.8) |
| **HI** | 44.4 (3.5) | 37.4 (4.0) | 52.8 (5.7) | 52.2 (5.7) | 44.7 (7.5) | 44.5 (4.6) | 46.3 (4.4) | 39.7 (4.4) | 56.4 (5.1) | 53.0 (5.9) | 47.4 (7.2) | 44.2 (5.5) |
| **NHI** | 82.0 (2.6) | 78.2 (4.1) | 89.2 (2.5) | 82.5 (4.6) | 86.4 (4.4) | 81.3 (4.3) | 82.4 (4.2) | 81.3 (4.2) | 89.3 (2.5) | 83.6 (4.3) | 85.0 (4.7) | 73.1 (6.8) |
| **NUE** | 32.2 (4.2) | 35.6 (5.5) | 33.3 (4.0) | 31.2 (4.7) | 36.1 (3.5) | 30.0 (3.8) | 28.6 (4.3) | 29.3 (4.9) | 32.0 (3.9) | 28.7 (4.3) | 28.9 (2.7) | 28.4 (3.6) |
| **NUpE** | 0.77 (0.09) | 0.73 (0.10) | 0.81 (0.07) | 0.76 (0.10) | 0.80 (0.07) | 0.80 (0.08) | 0.79 (0.10) | 0.72 (0.11) | 0.86 (0.09) | 0.81 (0.10) | 0.81 (0.08) | 0.80 (0.08) |
| **NUtE** | 42.1 (3.7) | 48.7 (4.9) | 40.9 (3.7) | 41.2 (4.1) | 45.5 (4.2) | 37.9 (4.5) | 36.2 (3.2) | 40.5 (4.4) | 37.3 (3.3) | 35.7 (3.9) | 36.0 (3.5) | 36.3 (5.5) |
| **NUtE_PROT** | 0.07 (0.03) | 0.11 (0.06) | 0.08 (0.01) | 0.09 (0.02) | 0.09 (0.01) | 0.07 (0.01) | 0.06 (0.01) | 0.11 (0.02) | 0.07 (0.01) | 0.08 (0.01) | 0.07 (0.01) | 0.06 (0.01) |
| **NUE_PROT** | 0.05 (0.00) | 0.08 (0.01) | 0.06 (0.01) | 0.07 (0.01) | 0.07 (0.01) | 0.05 (0.01) | 0.05 (0.00) | 0.07 (0.01) | 0.06 (0.01) | 0.06 (0.01) | 0.06 (0.00) | 0.05 (0.01) |
| **NRE** | 57.8 (11.0) | 48.7 (12.5) | 82.7 (6.6) | 64.1 (13.3) | 85.7 (7.8) | 52.3 (9.0) | 68.0 (8.5) | 61.7 (10.0) | 86.2 (4.1) | 72.1 (10.0) | 85.6 (7.3) | 39.6 (9.6) |
| **BPE** | 96.3 (10.7) | 152.1 (18.1) | 83.3 (10.5) | 81.2 (8.2) | 99.5 (21.6) | 114.9 (10.3) | 85.0 (9.2) | 125.9 (12.5) | 69.5 (6.0) | 67.6 (6.0) | 75.2 (9.6) | 101.7 (9.2) |
| **PANU** | 93.2 (21.0) | 42.3 (13.0) | 47.2 (13.6) | 65.3 (51.5) | 40.7 (48.1) | 68.7 (23.5) | 74.7 (29.2) | 43.0 (14.2) | 44.6 (14.8) | 71.4 (25.4) | 48.4 (30.8) | 75.2 (25.2) |
