# Supplementary material for: Variation for Nitrogen Use Efficiency Traits in Wheat Under Contrasting Nitrogen Treatments in South-Eastern Europe
Source: Front Plant Sci. 2021 Nov 18;12:682333. doi: 10.3389/fpls.2021.682333 (PMC8636685; doi:10.3389/fpls.2021.682333)
Supplement: Supplementary File 1 — (Meteo data 2016–2018) | The meteorological data file contains measurements taken at three weather stations of experimental sites (Osijek, Poreč, and Zagreb) during three consecutive years (2016, 2017 and 2018), and includes the information on average daily air temperatures (Sheet1), precipitations (Sheet 2), and summary values (Sheet 3). [file Data_Sheet_1.zip › Supplementary File 3_Supplementary Figures 1-3.pptx]

## Slide 1
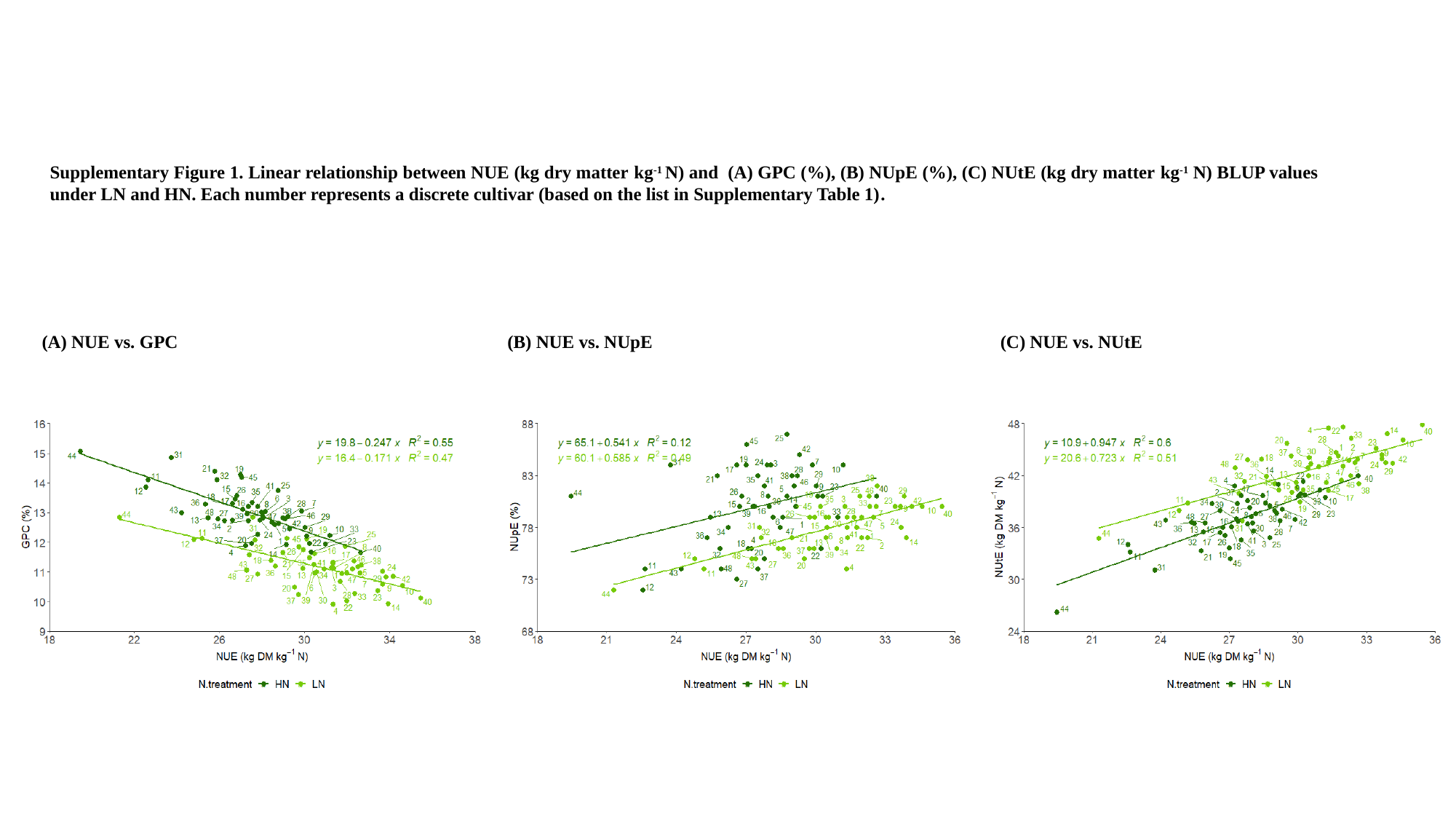

Supplementary Figure 1. Linear relationship between NUE (kg dry matter kg-1 N) and (A) GPC (%), (B) NUpE (%), (C) NUtE (kg dry matter kg-1 N) BLUP values under LN and HN. Each number represents a discrete cultivar (based on the list in Supplementary Table 1).
(A) NUE vs. GPC
(B) NUE vs. NUpE
(C) NUE vs. NUtE

## Slide 2
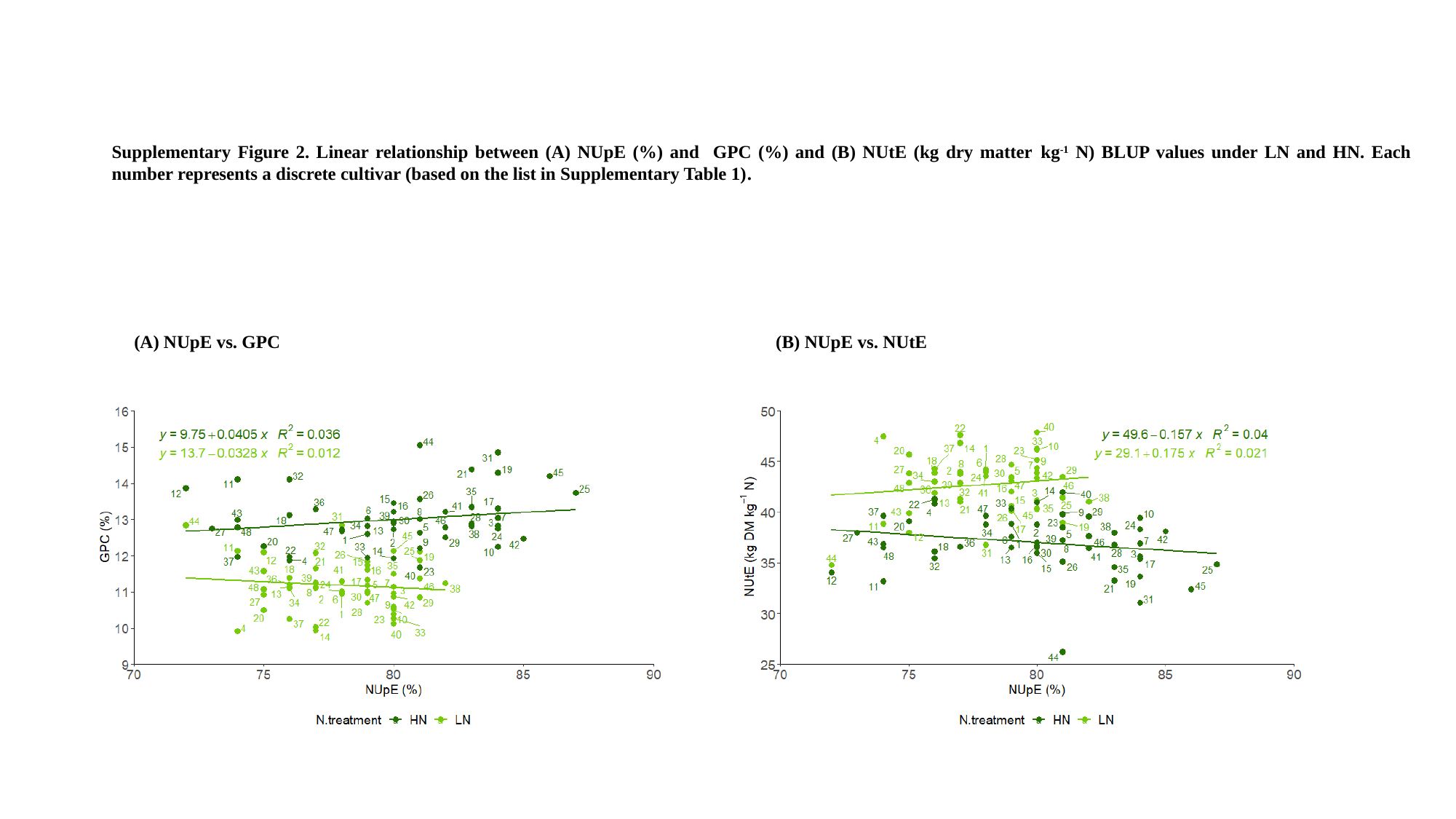

Supplementary Figure 2. Linear relationship between (A) NUpE (%) and GPC (%) and (B) NUtE (kg dry matter kg-1 N) BLUP values under LN and HN. Each number represents a discrete cultivar (based on the list in Supplementary Table 1).
(A) NUpE vs. GPC
(B) NUpE vs. NUtE

## Slide 3
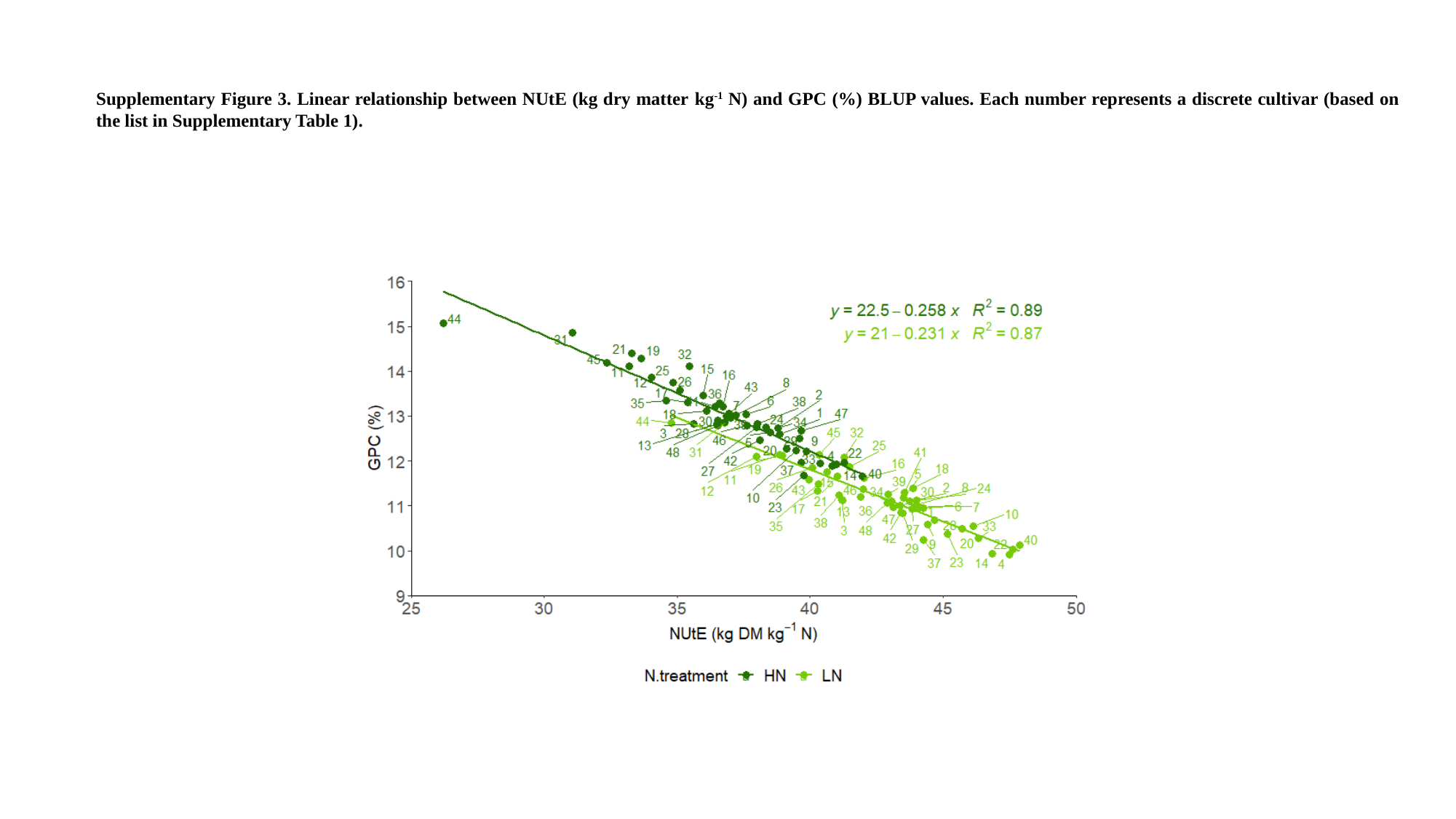

Supplementary Figure 3. Linear relationship between NUtE (kg dry matter kg-1 N) and GPC (%) BLUP values. Each number represents a discrete cultivar (based on the list in Supplementary Table 1).
