# Supplementary material for: Variation for Nitrogen Use Efficiency Traits in Wheat Under Contrasting Nitrogen Treatments in South-Eastern Europe
Source: Front Plant Sci. 2021 Nov 18;12:682333. doi: 10.3389/fpls.2021.682333 (PMC8636685; doi:10.3389/fpls.2021.682333)
Supplement: Supplementary File 1 — (Meteo data 2016–2018) | The meteorological data file contains measurements taken at three weather stations of experimental sites (Osijek, Poreč, and Zagreb) during three consecutive years (2016, 2017 and 2018), and includes the information on average daily air temperatures (Sheet1), precipitations (Sheet 2), and summary values (Sheet 3). [file Data_Sheet_1.zip › Supplementary File 4_Linear correlations.docx]

**Supplementary Table LC1. Linear correlations (r) between analyzed traits at low (LN) and high (HN) nitrogen levels in Osijek in 2017 (upper right triangle ) and 2018 (lower left triangle) year (n=48 cultivars).**

|  | N  level | GY | PH | GPC | GNY | NTA | HI | NHI | NUE | NUpE | NUtE | NUtE_ PROT | NUE_ PROT | NRE | BPE | PANU |
| --- | --- | --- | --- | --- | --- | --- | --- | --- | --- | --- | --- | --- | --- | --- | --- | --- |
| GY | LN |  | -0.44^**^ | -0.51^**^ | 0.79^**^ | 0.76^**^ | 0.62^**^ | 0.27 | 1.00^**^ | 0.77^**^ | 0.56^**^ | -0.56^**^ | -0.40^**^ | -0.09 | -0.43^**^ | 0.60^**^ |
|  | HN |  | -0.53^**^ | -0.39^**^ | 0.90^**^ | 0.77^**^ | 0.54^**^ | 0.35^*^ | 1.00^**^ | 0.77^**^ | 0.47^**^ | -0.89^**^ | -0.48^**^ | 0.02 | -0.46^**^ | 0.67^**^ |
| PH | LN | -0.61^**^ |  | 0.29^*^ | -0.34^*^ | -0.22 | -0.74^**^ | -0.58^**^ | -0.44^**^ | -0.22 | -0.44^**^ | 0.06 | 0.16 | -0.16 | 0.28 | -0.27 |
|  | HN | -0.64^**^ |  | 0.24 | -0.48^**^ | -0.30^*^ | -0.77^**^ | -0.36^*^ | -0.53^**^ | -0.30^*^ | -0.34^*^ | 0.31^*^ | 0.37^**^ | 0.03 | 0.71^**^ | -0.50^**^ |
| GPC | LN | -0.63^**^ | 0.41^**^ |  | 0.11 | 0.12 | -0.37^*^ | -0.09 | -0.51^**^ | 0.11 | -0.93^**^ | 0.07 | 0.76^**^ | 0.08 | -0.34^*^ | -0.12 |
|  | HN | -0.66^**^ | 0.48^**^ |  | 0.05 | 0.15 | -0.46^**^ | -0.16 | -0.39^**^ | 0.15 | -0.85^**^ | 0.33^*^ | 0.74^**^ | 0.16 | -0.30^*^ | -0.06 |
| GNY | LN | 0.88^**^ | -0.57^**^ | -0.19 |  | 0.97^**^ | 0.48^**^ | 0.29^*^ | 0.79^**^ | 0.97^**^ | 0.00 | -0.57^**^ | 0.08 | -0.03 | -0.74^**^ | 0.61^**^ |
|  | HN | 0.86^**^ | -0.57^**^ | -0.18 |  | 0.90^**^ | 0.39^**^ | 0.34^*^ | 0.90^**^ | 0.90^**^ | 0.12 | -0.80^**^ | -0.17 | 0.10 | -0.64^**^ | 0.70^**^ |
| NTA | LN | 0.74^**^ | -0.39^**^ | -0.05 | 0.90^**^ |  | 0.32^*^ | 0.04 | 0.76^**^ | 1.00^**^ | -0.10 | -0.59^**^ | 0.05 | -0.15 | -0.72^**^ | 0.64^**^ |
|  | HN | 0.71^**^ | -0.37^**^ | -0.02 | 0.91^**^ |  | 0.04 | -0.11 | 0.77^**^ | 1.00^**^ | -0.19 | -0.82^**^ | -0.05 | -0.09 | -0.60^**^ | 0.67^**^ |
| HI | LN | 0.63^**^ | -0.76^**^ | -0.57^**^ | 0.48^**^ | 0.17 |  | 0.71^**^ | 0.62^**^ | 0.33^*^ | 0.57^**^ | -0.19 | -0.30^*^ | 0.12 | -0.33^*^ | 0.30^*^ |
|  | HN | 0.68^**^ | -0.79^**^ | -0.57^**^ | 0.52^**^ | 0.19 |  | 0.74^**^ | 0.54^**^ | 0.04 | 0.73^**^ | -0.26 | -0.56^**^ | 0.13 | -0.38^**^ | 0.33^*^ |
| NHI | LN | 0.33^*^ | -0.46^**^ | -0.28 | 0.28 | -0.17 | 0.69^**^ |  | 0.27 | 0.03 | 0.43^**^ | -0.02 | 0.08 | 0.43^**^ | -0.18 | 0.05 |
|  | HN | 0.44^**^ | -0.50^**^ | -0.42^**^ | 0.31^*^ | -0.12 | 0.79^**^ |  | 0.35^*^ | -0.11 | 0.66^**^ | -0.03 | -0.25 | 0.50^**^ | -0.11 | 0.10 |
| NUE | LN | 1.00^**^ | -0.61^**^ | -0.63^**^ | 0.88^**^ | 0.74^**^ | 0.63^**^ | 0.33* |  | 0.77^**^ | 0.56^**^ | -0.56^**^ | -0.40^**^ | -0.09 | -0.43^**^ | 0.60^**^ |
|  | HN | 1.00^**^ | -0.64^**^ | -0.66^**^ | 0.86^**^ | 0.71^**^ | 0.68^**^ | 0.44^**^ |  | 0.77^**^ | 0.47^**^ | -0.89^**^ | -0.48^**^ | 0.02 | -0.46^**^ | 0.67^**^ |
| NUpE | LN | 0.74^**^ | -0.38^**^ | -0.05 | 0.89^**^ | 1.00^**^ | 0.16 | -0.18 | 0.74^**^ |  | -0.10 | -0.59^**^ | 0.04 | -0.15 | -0.72^**^ | 0.64^**^ |
|  | HN | 0.71^**^ | -0.37^*^ | -0.03 | 0.91^**^ | 1.00^**^ | 0.19 | -0.12 | 0.71^**^ |  | -0.19 | -0.82^**^ | -0.05 | -0.09 | -0.60^**^ | 0.67^**^ |
| NUtE | LN | 0.60^**^ | -0.47^**^ | -0.87^**^ | 0.24 | -0.09 | 0.74^**^ | 0.71^**^ | 0.60^**^ | -0.10 |  | -0.08 | -0.66^**^ | 0.08 | 0.26 | 0.12 |
|  | HN | 0.66^**^ | -0.52^**^ | -0.91^**^ | 0.25 | -0.06 | 0.74^**^ | 0.74^**^ | 0.66^**^ | -0.06 |  | -0.25 | -0.70^**^ | 0.16 | 0.20 | 0.08 |
| NUtE_PROT | LN | -0.93^**^ | 0.58^**^ | 0.55^**^ | -0.85^**^ | -0.83^**^ | -0.48^**^ | -0.09 | -0.93^**^ | -0.82^**^ | -0.41^**^ |  | 0.11 | 0.21 | 0.73^**^ | -0.21 |
|  | HN | -0.89^**^ | 0.46^**^ | 0.53^**^ | -0.80^**^ | -0.81^**^ | -0.38^**^ | -0.07 | -0.89^**^ | -0.81^**^ | -0.41^**^ |  | 0.40^**^ | 0.09 | 0.37^*^ | -0.57^**^ |
| NUE_PROT | LN | -0.56^**^ | 0.58^**^ | 0.87^**^ | -0.17 | -0.04 | -0.54^**^ | -0.24 | -0.56^**^ | -0.03 | -0.76^**^ | 0.49^**^ |  | 0.13 | -0.20 | -0.13 |
|  | HN | -0.45^**^ | 0.46^**^ | 0.84^**^ | 0.00 | 0.13 | -0.42^**^ | -0.34^*^ | -0.45^**^ | 0.13 | -0.77^**^ | 0.30^*^ |  | 0.10 | -0.09 | -0.19 |
| NRE | LN | 0.03 | -0.23 | -0.21 | -0.07 | -0.34^*^ | 0.36^*^ | 0.56^**^ | 0.03 | -0.35^*^ | 0.47^**^ | 0.12 | -0.17 |  | -0.02 | -0.59^**^ |
|  | HN | 0.18 | -0.23 | -0.40^**^ | -0.04 | -0.32^*^ | 0.43^**^ | 0.66^**^ | 0.18 | -0.33^*^ | 0.60^**^ | 0.07 | -0.35^*^ |  | -0.09 | -0.50^**^ |
| BPE | LN | -0.36^*^ | 0.62^**^ | -0.16 | -0.58^**^ | -0.60^**^ | -0.38^**^ | 0.00 | -0.36^*^ | -0.60^**^ | 0.17 | 0.44^**^ | -0.15 | -0.02 |  | -0.27 |
|  | HN | -0.34^*^ | 0.51^**^ | -0.25 | -0.65^**^ | -0.64^**^ | -0.35^*^ | -0.03 | -0.34^*^ | -0.63^**^ | 0.21 | 0.36^*^ | -0.42^**^ | 0.11 |  | -0.50^**^ |
| PANU | LN | 0.30* | -0.06 | 0.05 | 0.41^**^ | 0.52^**^ | -0.07 | -0.23 | 0.30^*^ | 0.52^**^ | -0.17 | -0.36^*^ | 0.15 | -0.33^*^ | -0.10 |  |
|  | HN | 0.34^*^ | -0.12 | 0.21 | 0.59^**^ | 0.69^**^ | 0.00 | -0.18 | 0.34^*^ | 0.69^**^ | -0.26 | -0.45^**^ | 0.22 | -0.72^**^ | -0.38^**^ |  |

*,** - Significance (df=46) at the level of probability *p* < 0.05 and *p* < 0.01, respectively.

**Supplementary Table LC2. Linear correlations (r) between analyzed traits at low (LN) and high (HN) nitrogen levels in Poreč in 2017 (upper right triangle ) and 2018 (lower left triangle) year (n=48 cultivars).**

|  | N  level | GY | PH | GPC | GNY | NTA | HI | NHI | NUE | NUpE | NUtE | NUtE_ PROT | NUE_ PROT | NRE | BPE | PANU |
| --- | --- | --- | --- | --- | --- | --- | --- | --- | --- | --- | --- | --- | --- | --- | --- | --- |
| GY | LN |  | -0.14 | -0.49^**^ | 0.86^**^ | 0.79^**^ | 0.27 | 0.15 | 1.00^**^ | 0.79^**^ | 0.46^**^ | -0.50^**^ | -0.39^**^ | 0.23 | -0.31^*^ | 0.32^*^ |
|  | HN |  | -0.17 | -0.49^**^ | 0.89^**^ | 0.79^**^ | 0.61^**^ | 0.44^**^ | 1.00^**^ | 0.80^**^ | 0.55^**^ | -0.88^**^ | -0.57^**^ | -0.12 | -0.30^*^ | 0.47^**^ |
| PH | LN | -0.35^*^ |  | 0.16 | -0.09 | 0.04 | -0.65^**^ | -0.36^*^ | -0.14 | 0.04 | -0.30^*^ | -0.11 | 0.18 | 0.08 | 0.31^*^ | -0.24 |
|  | HN | -0.54^**^ |  | 0.31^*^ | -0.05 | 0.08 | -0.56^**^ | -0.37^*^ | -0.17 | 0.07 | -0.38^**^ | 0.09 | 0.32^*^ | -0.13 | 0.29^*^ | -0.09 |
| GPC | LN | -0.59^**^ | 0.40^**^ |  | 0.02 | 0.03 | -0.51^**^ | -0.06 | -0.49^**^ | 0.02 | -0.84^**^ | 0.10 | 0.88^**^ | -0.07 | -0.19 | 0.01 |
|  | HN | -0.53^**^ | 0.41^**^ |  | -0.06 | 0.06 | -0.61^**^ | -0.45^**^ | -0.49^**^ | 0.06 | -0.90^**^ | 0.36^*^ | 0.88^**^ | -0.18 | -0.35^*^ | -0.04 |
| GNY | LN | 0.71^**^ | -0.14 | 0.14 |  | 0.93^**^ | 0.02 | 0.13 | 0.86^**^ | 0.93^**^ | 0.03 | -0.50^**^ | 0.05 | 0.22 | -0.47^**^ | 0.39^**^ |
|  | HN | 0.79^**^ | -0.38^**^ | 0.10 |  | 0.95^**^ | 0.39^**^ | 0.29^*^ | 0.89^**^ | 0.95^**^ | 0.16 | -0.85^**^ | -0.21 | -0.23 | -0.52^**^ | 0.49^**^ |
| NTA | LN | 0.44^**^ | -0.07 | 0.25 | 0.77^**^ |  | -0.21 | -0.25 | 0.79^**^ | 1.00^**^ | -0.18 | -0.55^**^ | 0.01 | 0.09 | -0.59^**^ | 0.39^**^ |
|  | HN | 0.16 | 0.07 | 0.60^**^ | 0.58^**^ |  | 0.17 | -0.02 | 0.79^**^ | 1.00^**^ | -0.06 | -0.81^**^ | -0.10 | -0.39^**^ | -0.59^**^ | 0.48^**^ |
| HI | LN | 0.36^*^ | -0.27 | -0.33^*^ | 0.17 | -0.43^**^ |  | 0.61^**^ | 0.27 | -0.20 | 0.73^**^ | 0.15 | -0.4^**^ | -0.05 | 0.11 | 0.16 |
|  | HN | 0.47^**^ | -0.39^**^ | -0.51^**^ | 0.21 | -0.44^**^ |  | 0.77^**^ | 0.61^**^ | 0.17 | 0.78^**^ | -0.40^**^ | -0.62^**^ | 0.14 | -0.10 | 0.27 |
| NHI | LN | 0.26 | -0.08 | -0.21 | 0.15 | -0.51^**^ | 0.92^**^ |  | 0.15 | -0.24 | 0.59^**^ | 0.20 | 0.09 | 0.37^*^ | 0.38^**^ | -0.05 |
|  | HN | 0.52^**^ | -0.34^*^ | -0.60^**^ | 0.22 | -0.63^**^ | 0.82^**^ |  | 0.44^**^ | -0.02 | 0.78^**^ | -0.27 | -0.41^**^ | 0.44^**^ | 0.24 | 0.11 |
| NUE | LN | 1.00^**^ | -0.35^*^ | -0.59^**^ | 0.71^**^ | 0.44^**^ | 0.36^*^ | 0.26 |  | 0.79^**^ | 0.46^**^ | -0.50^**^ | -0.39^**^ | 0.23 | -0.31^*^ | 0.32^*^ |
|  | HN | 1.00^**^ | -0.54^**^ | -0.53^**^ | 0.79^**^ | 0.16 | 0.47^**^ | 0.52^**^ |  | 0.80^**^ | 0.55^**^ | -0.88^**^ | -0.57^**^ | -0.12 | -0.30^*^ | 0.47^**^ |
| NUpE | LN | 0.44^**^ | -0.07 | 0.25 | 0.76^**^ | 1.00^**^ | -0.44^**^ | -0.51^**^ | 0.44^**^ |  | -0.17 | -0.56^**^ | 0.01 | 0.09 | -0.59^**^ | 0.39^**^ |
|  | HN | 0.16 | 0.07 | 0.60^**^ | 0.58^**^ | 1.00^**^ | -0.45^**^ | -0.64^**^ | 0.16 |  | -0.06 | -0.81^**^ | -0.10 | -0.39^**^ | -0.59^**^ | 0.48^**^ |
| NUtE | LN | 0.58^**^ | -0.31^*^ | -0.84^**^ | -0.02 | -0.46^**^ | 0.73^**^ | 0.69^**^ | 0.58^**^ | -0.46^**^ |  | 0.02 | -0.66^**^ | 0.26 | 0.37^*^ | -0.07 |
|  | HN | 0.59^**^ | -0.39^**^ | -0.91^**^ | 0.04 | -0.68^**^ | 0.74^**^ | 0.87^**^ | 0.59^**^ | -0.68^**^ |  | -0.36^*^ | -0.80^**^ | 0.33^*^ | 0.37^**^ | 0.12 |
| NUtE_PROT | LN | -0.74^**^ | 0.23 | 0.34^*^ | -0.61^**^ | -0.74^**^ | 0.17 | 0.31^*^ | -0.73^**^ | -0.75^**^ | -0.08 |  | 0.05 | -0.04 | 0.65^**^ | -0.22 |
|  | HN | -0.73^**^ | 0.41^**^ | 0.18 | -0.71^**^ | -0.57^**^ | 0.01 | 0.07 | -0.75^**^ | -0.57^**^ | -0.07 |  | 0.41^**^ | 0.29^*^ | 0.33^*^ | -0.37^*^ |
| NUE_PROT | LN | -0.50^**^ | 0.44^**^ | 0.84^**^ | 0.11 | 0.17 | -0.27 | -0.11 | -0.50^**^ | 0.17 | -0.67^**^ | 0.29^*^ |  | 0.06 | -0.12 | -0.06 |
|  | HN | -0.35^*^ | 0.19 | 0.72^**^ | 0.11 | 0.43^**^ | -0.29^*^ | -0.37^**^ | -0.35^*^ | 0.43^**^ | -0.63^**^ | 0.15 |  | -0.15 | -0.13 | -0.14 |
| NRE | LN | 0.37^*^ | -0.40^**^ | -0.38^**^ | 0.14 | -0.09 | 0.43^**^ | 0.33^*^ | 0.37^*^ | -0.08 | 0.44^**^ | -0.17 | -0.36^*^ |  | 0.16 | -0.61^**^ |
|  | HN | 0.37^*^ | -0.36^*^ | -0.28 | 0.24 | -0.08 | 0.27 | 0.32^*^ | 0.37^*^ | -0.09 | 0.33^*^ | -0.19 | -0.08 |  | 0.26 | -0.56^**^ |
| BPE | LN | 0.06 | 0.12 | -0.06 | -0.04 | 0.12 | 0.43^**^ | -0.26 | 0.06 | 0.12 | -0.04 | -0.07 | -0.04 | -0.14 |  | -0.34^*^ |
|  | HN | -0.36^*^ | 0.39^**^ | -0.13 | -0.52^**^ | -0.31^*^ | 0.27 | -0.16 | -0.36^*^ | -0.30^*^ | -0.01 | 0.36^*^ | -0.26 | -0.42^**^ |  | -0.21 |
| PANU | LN | 0.18 | 0.02 | -0.08 | 0.14 | 0.03 | 0.10 | 0.14 | 0.18 | 0.03 | 0.13 | -0.09 | -0.14 | -0.01 | -0.02 |  |
|  | HN | -0.17 | 0.05 | 0.19 | -0.05 | 0.00 | -0.10 | -0.07 | -0.17 | 0.00 | -0.16 | 0.10 | -0.05 | -0.49^**^ | -0.06 |  |

*,** - Significance (df=46) at the level of probability *p* < 0.05 and *p* < 0.01, respectively.

**Supplementary Table LC3. Linear correlations (r) between analyzed traits at low (LN) and high (HN) nitrogen levels in Zagreb in 2017 (upper right triangle ) and 2018 (lower left triangle) year (n=48 cultivars).**

|  | N  level | GY | PH | GPC | GNY | NTA | HI | NHI | NUE | NUpE | NUtE | NUtE_ PROT | NUE_ PROT | NRE | BPE | PANU |
| --- | --- | --- | --- | --- | --- | --- | --- | --- | --- | --- | --- | --- | --- | --- | --- | --- |
| GY | LN |  | -0.26 | -0.63^**^ | 0.79^**^ | 0.68^**^ | 0.58^**^ | 0.52^**^ | 1.00^**^ | 0.68^**^ | 0.67^**^ | -0.92^**^ | -0.44^**^ | 0.72^**^ | -0.46^**^ | -0.15 |
|  | HN |  | -0.24 | -0.59^**^ | 0.82^**^ | 0.71^**^ | 0.50^**^ | 0.47^**^ | 1.00^**^ | 0.70^**^ | 0.62^**^ | -0.92^**^ | -0.38^**^ | 0.40^**^ | -0.24 | 0.26 |
| PH | LN | -0.70^**^ |  | 0.08 | -0.29^*^ | -0.24 | -0.38^**^ | -0.20 | -0.26 | -0.25 | -0.10 | 0.23 | 0.04 | 0.01 | 0.32^*^ | -0.08 |
|  | HN | -0.58^**^ |  | 0.11 | -0.23 | -0.18 | -0.51^**^ | -0.23 | -0.25 | -0.18 | -0.16 | 0.25 | 0.03 | -0.15 | 0.44^**^ | -0.08 |
| GPC | LN | -0.30^*^ | 0.42^**^ |  | -0.03 | 0.09 | -0.41^**^ | -0.39^**^ | -0.63^**^ | 0.09 | -0.95^**^ | 0.61^**^ | 0.83^**^ | -0.47^**^ | -0.12 | 0.21 |
|  | HN | -0.49^**^ | 0.37^*^ |  | -0.02 | 0.12 | -0.58^**^ | -0.50^**^ | -0.59^**^ | 0.12 | -0.96^**^ | 0.52^**^ | 0.87^**^ | -0.47^**^ | -0.32^*^ | 0.02 |
| GNY | LN | 0.57^**^ | -0.31^*^ | 0.21 |  | 0.95^**^ | 0.42^**^ | 0.36^*^ | 0.79^**^ | 0.95^**^ | 0.12 | -0.72^**^ | 0.07 | 0.55^**^ | -0.68^**^ | 0.01 |
|  | HN | 0.33^*^ | -0.49^**^ | 0.33^*^ |  | 0.96^**^ | 0.21 | 0.23 | 0.82^**^ | 0.96^**^ | 0.08 | -0.78^**^ | 0.16 | 0.17 | -0.53^**^ | 0.35^*^ |
| NTA | LN | 0.57^**^ | -0.25 | 0.29^*^ | 0.92^**^ |  | 0.18 | 0.07 | 0.68^**^ | 1.00^**^ | -0.07 | -0.67^**^ | 0.17 | 0.39^**^ | -0.62^**^ | 0.03 |
|  | HN | 0.08 | -0.07 | 0.51^**^ | 0.67^**^ |  | -0.02 | -0.05 | 0.71^**^ | 1.00^**^ | -0.12 | -0.71^**^ | 0.27 | -0.06 | -0.47^**^ | 0.34^*^ |
| HI | LN | 0.58^**^ | -0.72^**^ | -0.48^**^ | 0.35^*^ | 0.12 |  | 0.84^**^ | 0.58^**^ | 0.18 | 0.60^**^ | -0.40^**^ | -0.29^*^ | 0.51^**^ | -0.59^**^ | -0.10 |
|  | HN | 0.56^**^ | -0.69^**^ | -0.35^*^ | 0.40^**^ | -0.28 |  | 0.83^**^ | 0.50^**^ | -0.02 | 0.72^**^ | -0.41^**^ | -0.49^**^ | 0.63^**^ | -0.40^**^ | 0.09 |
| NHI | LN | 0.27 | -0.30^*^ | -0.14 | 0.57^**^ | 0.20 | 0.67^**^ |  | 0.52^**^ | 0.07 | 0.65^**^ | -0.30^*^ | -0.33^*^ | 0.63^**^ | -0.34^*^ | -0.09 |
|  | HN | 0.33^*^ | -0.55^**^ | -0.04 | 0.65^**^ | -0.12 | 0.81^**^ |  | 0.47^**^ | -0.05 | 0.71^**^ | -0.33^*^ | -0.36^*^ | 0.81^**^ | -0.25 | 0.05 |
| NUE | LN | 1.00^**^ | -0.70^**^ | -0.30^*^ | 0.57^**^ | 0.57^**^ | 0.58^**^ | 0.27 |  | 0.68^**^ | 0.67^**^ | -0.92^**^ | -0.44^**^ | 0.72^**^ | -0.46^**^ | -0.15 |
|  | HN | 1.00^**^ | -0.58^**^ | -0.49^**^ | 0.33^*^ | 0.08 | 0.56^**^ | 0.33^*^ |  | 0.71^**^ | 0.62^**^ | -0.92^**^ | -0.38^**^ | 0.40^**^ | -0.24 | 0.26 |
| NUpE | LN | 0.57^**^ | -0.25 | 0.28 | 0.92^**^ | 1.00^**^ | 0.12 | 0.21 | 0.57^**^ |  | -0.08 | -0.67^**^ | 0.17 | 0.38^**^ | -0.62^**^ | 0.03 |
|  | HN | 0.08 | -0.07 | 0.51^**^ | 0.67^**^ | 1.00^**^ | -0.27 | -0.11 | 0.08 |  | -0.12 | -0.71^**^ | 0.26 | -0.06 | -0.47^**^ | 0.34^*^ |
| NUtE | LN | 0.60^**^ | -0.57^**^ | -0.63^**^ | -0.22 | -0.31^*^ | 0.57^**^ | 0.14 | 0.60^**^ | -0.31^*^ |  | -0.58^**^ | -0.77^**^ | 0.60^**^ | 0.02 | -0.23 |
|  | HN | 0.74^**^ | -0.36^*^ | -0.71^**^ | -0.22 | -0.60^**^ | 0.59^**^ | 0.30^*^ | 0.74^**^ | -0.59^**^ |  | -0.51^**^ | -0.83^**^ | 0.62^**^ | 0.19 | 0.01 |
| NUtE_PROT | LN | -0.71^**^ | 0.59^**^ | 0.44^**^ | -0.68^**^ | -0.66^**^ | -0.51^**^ | -0.36^*^ | -0.71^**^ | -0.68^**^ | -0.18 |  | 0.41^**^ | -0.56^**^ | 0.36^*^ | 0.10 |
|  | HN | -0.57^**^ | 0.54^**^ | 0.44^**^ | -0.39^**^ | -0.46^**^ | -0.15 | -0.04 | -0.57^**^ | -0.46^**^ | -0.13 |  | 0.32^*^ | -0.24 | 0.26 | -0.36^*^ |
| NUE_PROT | LN | -0.20 | 0.27 | 0.83^**^ | 0.20 | 0.28 | -0.32^*^ | -0.12 | -0.20 | 0.27 | -0.51^**^ | 0.33^*^ |  | -0.34^*^ | -0.15 | 0.15 |
|  | HN | -0.43^**^ | 0.27 | 0.87^**^ | 0.34^*^ | 0.45^**^ | -0.27 | 0.03 | -0.43^**^ | 0.45^**^ | -0.61^**^ | 0.35^*^ |  | -0.33^*^ | -0.38^**^ | -0.03 |
| NRE | LN | 0.02 | 0.03 | -0.01 | 0.01 | -0.15 | 0.02 | 0.32^*^ | 0.02 | -0.16 | 0.16 | 0.04 | -0.04 |  | -0.47^**^ | -0.36^*^ |
|  | HN | 0.26 | -0.20 | -0.13 | 0.13 | -0.23 | 0.26 | 0.44^**^ | 0.26 | -0.23 | 0.35^*^ | -0.03 | -0.13 |  | -0.23 | -0.25 |
| BPE | LN | -0.21 | 0.27 | -0.49^**^ | -0.43^**^ | -0.60^**^ | -0.01 | 0.18 | -0.21 | -0.60^**^ | 0.36^*^ | 0.22 | -0.53^**^ | 0.13 |  | -0.07 |
|  | HN | 0.17 | 0.25 | -0.52^**^ | -0.50^**^ | -0.67^**^ | 0.02 | 0.05 | 0.17 | -0.67^**^ | 0.62^**^ | 0.16 | -0.45^**^ | 0.27 |  | -0.09 |
| PANU | LN | 0.37^*^ | -0.26 | 0.17 | 0.68^**^ | 0.68^**^ | 0.23 | 0.27 | 0.37^*^ | 0.69^**^ | -0.21 | -0.44^**^ | 0.16 | -0.64^**^ | -0.31^*^ |  |
|  | HN | 0.10 | -0.28 | 0.16 | 0.51^**^ | 0.53^**^ | 0.10 | 0.11 | 0.10 | 0.53^**^ | -0.29^*^ | -0.37^*^ | 0.25 | -0.64^**^ | -0.42^**^ |  |

*,** - Significance (df=46) at the level of probability *p* < 0.05 and *p* < 0.01, respectively.
